# Improving hematopoietic differentiation from human induced pluripotent stem cells by the modulation of Hippo signaling with a diarylheptanoid derivative

**DOI:** 10.1186/s13287-024-03686-4

**Published:** 2024-03-03

**Authors:** Umnuaychoke Thongsa-ad, Anongnat Wongpan, Wasinee Wongkummool, Phaewa Chaiwijit, Kwanchanok Uppakara, Gorawin Chaiyakitpattana, Passanan Singpant, Pirut Tong-ngam, Amnat Chukhan, Wachirachai Pabuprappap, Sirapope Wongniam, Apichart Suksamrarn, Suradej Hongeng, Usanarat Anurathapan, Kasem Kulkeaw, Alisa Tubsuwan, Kanit Bhukhai

**Affiliations:** 1 Department of Physiology, Faculty of Science, Mahidol University, Bangkok, 10400 Thailand; 2 Stem Cell Research Group, Institute of Molecular Biosciences, Mahidol University, Nakhon Pathom, 73170 Thailand; 3 Center of Multidisciplinary Technology for Advanced Medicine (CMUTEAM), Faculty of Medicine, Chiang Mai University, Chiang Mai, 50200 Thailand; 4 Chakri Naruebodindra Medical Institute, Faculty of Medicine Ramathibodi Hospital, Mahidol University, Samut Prakan, 10540 Thailand; 5 Prima Scientific, 147/170-171 Baromrajchonnee, Arunamarin, Bangkok, 10700 Thailand; 6 Department of Chemistry and Center of Excellence for Innovation in Chemistry, Faculty of Science, Ramkhamhaeng University, Bangkok, 10240 Thailand; 7 Center for Scientific Instrumentation and Platform Services Unit, Faculty of Science, Mahidol University, Bangkok, 10400 Thailand; 8 Department of Pediatrics, Faculty of Medicine, Ramathibodi Hospital, Mahidol University, Bangkok, 10400 Thailand; 9 Siriraj Integrative Center for Neglected Parasitic Diseases, Department of Parasitology, Faculty of Medicine Siriraj Hospital, Mahidol University, Bangkok, 10700 Thailand

**Keywords:** Diarylheptanoid, Human induced pluripotent stem cells, Primitive hematopoietic stem and progenitor cells, Hippo signaling pathway

## Abstract

**Background:**

The diarylheptanoid ASPP 049 has improved the quality of adult hematopoietic stem cell (HSC) expansion ex vivo through long-term reconstitution in animal models. However, its effect on hematopoietic regeneration from human induced pluripotent stem cells (hiPSCs) is unknown.

**Method:**

We utilized a defined cocktail of cytokines without serum or feeder followed by the supplementation of ASPP 049 to produce hematopoietic stem/progenitor cells (HSPCs). Flow cytometry and trypan blue exclusion analysis were used to identify nonadherent and adherent cells. Nonadherent cells were harvested to investigate the effect of ASPP 049 on multipotency using LTC-IC and CFU assays. Subsequently, the mechanism of action was explored through transcriptomic profiles, which were validated by qRT-PCR, immunoblotting, and immunofluorescence analysis.

**Result:**

The supplementation of ASPP 049 increased the number of phenotypically defined primitive HSPCs (CD34^+^CD45^+^CD90^+^) two-fold relative to seeded hiPSC colonies, indicating enhanced HSC derivation from hiPSCs. Under ASPP 049-supplemented conditions, we observed elevated HSPC niches, including CD144^+^CD73^−^ hemogenic- and CD144^+^CD73^+^ vascular-endothelial progenitors, during HSC differentiation. Moreover, harvested ASPP 049-treated cells exhibited improved self-renewal and a significantly larger proportion of different blood cell colonies with unbiased lineages, indicating enhanced HSC stemness properties. Transcriptomics and KEGG analysis of sorted CD34^+^CD45^+^ cells-related mRNA profiles revealed that the Hippo signaling pathway is the most significant in responding to WWTR1/TAZ, which correlates with the validation of the protein expression. Interestingly, ASPP 049-supplemented HSPCs upregulated 11 genes similarly to umbilical cord blood-derived HSPCs.

**Conclusion:**

These findings suggest that ASPP 049 can improve HSC-generating protocols with proliferative potentials, self-renewal ability, unbiased differentiation, and a definable mechanism of action for the clinical perspective of hematopoietic regenerative medicine.

**Graphical abstract:**

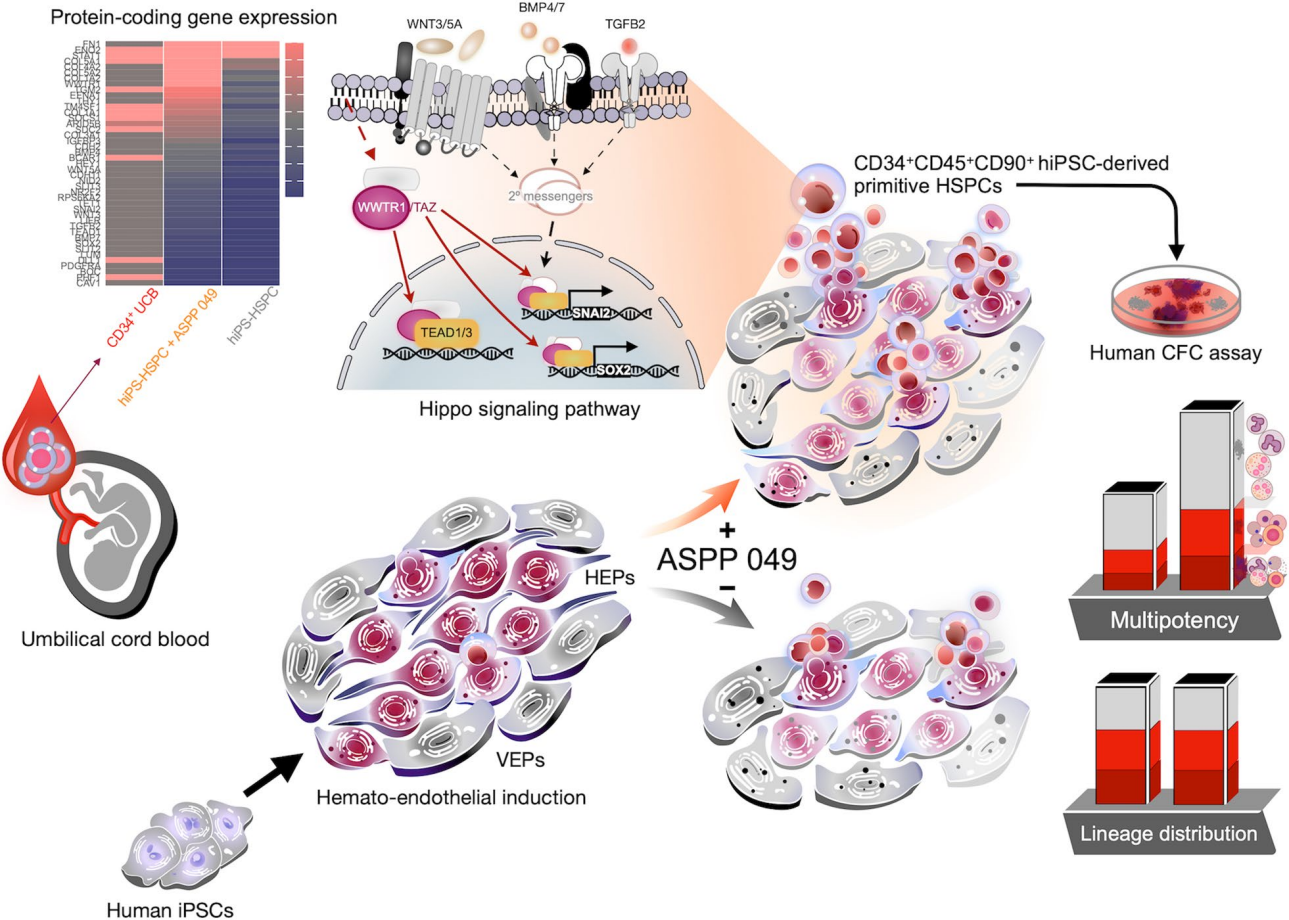

**Supplementary Information:**

The online version contains supplementary material available at 10.1186/s13287-024-03686-4.

## Background

Human induced pluripotent stem cells (hiPSCs) generated in vitro from adult cells have been offered promising potentials for improved proliferation, self-renewal, and pluripotent differentiation [1, 2]. Comparable to human embryonic stem cells (hESCs), hiPSCs can differentiate into vital cell types such as hematopoietic stem cells (HSCs), providing opportunities for treating hematological disorders through autologous HSC-based therapy [3, 4]. However, attempts to generate HSCs using a defined protocol have needed to be completed [5–7]. Generating HSCs from hiPSCs-directed differentiation protocols mainly involved cytokine cocktails that mimic mesodermal lineage commitment and endothelial progenitor with a hemogenic specification [8–11]. Hemogenic endothelial progenitors (HEPs) undergo the endothelial-to-hematopoietic transition (EHT) to generate hematopoietic stem/progenitor cells (HSPCs) [12–14]. One challenge with hiPSC-derived HSPCs is that their characteristics differ from those of HSPCs from other clinical sources [15, 16]. To overcome these limitations, clearly defined factors, including culture media, recombinant cytokines, and specific small molecules, regulate HSC differentiation precisely [8, 17–19].

Several studies have shown that small molecules and natural derivatives can enhance the hematopoietic cell fate of stem cells and improve HSC generation by increasing HSC number and functions [20–22]. A small molecule, StemRegnin 1, can promote the proliferation of CD34^+^CD45^+^ hESC/hiPSC-derived HSPCs. However, activating the aryl hydrocarbon receptor pathway can lead to accelerated natural killer (NK) cell differentiation or an imbalance in erythroid-megakaryocytic potentials [23, 24]. Similarly, UM171 showed an effect on hESC-derived hematopoietic cells after differentiation, which amplified CD34^+^CD41a^lo^CD45^+^ and CD34^+^CD45^+^CD7^+^ progenitors under HSC expansion and lymphoid induction, respectively. However, granulocytic enrichment and NK cell amplification were observed [25]. Likewise, a histone deacetylase inhibitor, suberoylanilide hydroxamic acid, increases CD34^+^CD45^+^ cells from embryoid bodies-derived HEP and predominantly drives the expression of platelet-related genes within HSPCs committed to premature megakaryopoiesis [26]. These observations illustrate multiple mechanisms impacting HSC differentiation systems. Therefore, identifying a novel small molecule that enhances hiPSC-derived HSPCs while preserving HSC capacities would be useful for in vitro HSC generation.

Previous studies have revealed the constructive effects of ASPP 049, a diarylheptanoid isolated from *Curcuma comosa* [27, 28], on an ex vivo CD34^+^CD38^−^CD90^+^ human HSC-culturing system. This compound potentially induces the cultured HSCs to have multi-lineage differentiation abilities in vitro, long-term reconstitution in vivo [29], as well as hematopoiesis improvement in an anemic mouse model [30]. However, the potential of HSC derivation from hiPSCs has not been investigated. This study aimed to explore the capability of ASPP 049 to affect HSC generation. We demonstrated that ASPP 049, via the Hippo signaling pathway, increased the quantity of CD34^+^CD45^+^ hiPSC-derived HSPCs with unbiased lineage distribution and preserved biological function. Furthermore, HSPCs generated under ASPP 049-supplemented conditions exhibited gene expression profiles similar to UCB-derived CD34^+^ cells. These findings suggest that ASPP 049 could be a novel component in the development of improved differentiation protocols for hiPSCs to generate HSCs.

## Methods

### Cell maintenance

hiPSC line, MUi019-A, was derived from CD34^+^ HSPCs from a healthy donor, as previously described [31]. The hiPSCs were maintained on Matrigel-coated dishes (Corning, VWR International) in Essential 8 (E8) medium supplemented with 1% Penicillin/Streptomycin (P/S, Gibco) under 5% O_2_ and 5% CO_2_ at 37°C with 95% humidity. The hiPSCs were replenished daily with E8 medium. When hiPSCs reached 70–80% confluence, they were passaged with 0.5 mM EDTA (ThermoFisher Scientific/Sigma) for 5 min. The colonies were dissociated into aggregates (10–100 cells) and passaged by ten-fold dilution in E8 medium supplemented with 10 mM Rho-associated coiled-coil-containing protein kinase inhibitor (ROCKi) (Y-27632, Sigma) for the first day of culture. The hiPSCs were experimentally used within passage numbers ranging from 20 to 30. Murine stromal fibroblasts, AFT024 cells, used for long-term culture (LTC) in limiting dilution analysis (LDA) of stem cells, were obtained from ATCC (SCRC-1007). The cells were seeded and expanded in 75 cm^2^ culture flask with Dulbecco’s Modified Eagle Medium, high-glucose, pyruvate (Gibco) containing 10% fetal bovine serum (FBS, Hyclone), 1% P/S and 50 μM β-mercaptoethanol (2-ME, ThermoFisher Scientific) under 5% CO_2_ at 33 °C with 95% humidity. AFT024 cells were experimentally used within passage numbers ranging from 1 to 2.

### Hematopoietic cell differentiation

hiPSC colonies were passaged and seeded as aggregates on a Matrigel-coated plate at a density of 10–15% confluency per well in a 12-well plate. Subsequently, E8 medium supplemented with ROCKi was added before differentiation into hematopoietic cells (Day 0). After 24 h, the medium was replaced with STEMdiff™ APEL2 (STEMCELL Technologies) media supplemented with 1% P/S and stepwise cytokines for hematopoietic induction with or without ASPP 049 (the processes of isolation and purification were previously described) [27, 28]. Briefly, the cells were induced by the addition of recombinant human BMP4 (50 ng/mL, PeproTech) for two days, followed by the addition of recombinant human VEGF (50 ng/mL, PeproTech) and recombinant human bFGF (50 ng/mL, PeproTech) from day 3 to 5. On day 5, in addition to VEGF and bFGF, SB431542 (20 μM, Sigma) was added. On day 7, the cytokines in the medium were substituted with recombinant human SCF (50 ng/mL, PeproTech), recombinant human IL-3 (50 ng/mL, PeproTech), and recombinant human TPO (50 ng/mL, PeproTech). During the following five days, the cells were treated with ASPP 049 and DMSO vehicle control in APEL2 media supplemented with the day 7 cytokine cocktail, and half-volume media was changed every other day. The differentiation culture was maintained at 37ºC with 5% CO_2_ and 5% O_2_ in a standard 95% humidified incubator.

### Cell number and viability assay

Cell viability was assessed using the Trypan blue exclusion assay. Total cell number and viability were determined using a hemocytometer, and the observation was recorded under a bright-field microscope.

### Flow cytometry analysis

At desired time points, nonadherent and adherent cells were harvested separately by aspiration and dissociation with 0.5 mM EDTA for 15 min, respectively. The cells were then filtered through 40 μm strainers, pelleted, and stained with antibodies in PBE buffer (PBS + 0.5% BSA + 2 mM EDTA). Antibodies of flow cytometry were used as follows: PerCP-conjugated CD34 (BioLegend), FITC-conjugated CD45 (BD Biosciences), and APC-conjugated CD90 (BioLegend), PE-conjugated CD144 (BD Biosciences), and APC-conjugated CD73 (BD Biosciences). Detailed information on these antibodies is provided in Additional file 1: Table S1. All samples were analyzed using BD Accuri™ C6 (BD Biosciences) and Attune™ Nxt (ThermoFisher Scientific). Cell sorting was performed using a FACSAria™ III (BD Biosciences). The data of flow cytometry was analyzed using Flowjo software.

### MyeloCult™ LTC-initiating cell (LTC-IC) for limiting dilution analysis (LDA)

AFT024 cells were seeded and grown overnight in 0.1% gelatin-coated 96-well plates at a density of 3–3.5 × 10^5^ cells per mL in the AFT024 cell-expanded condition as previously described [32]. Before 16 h of LTC, stromal cells were mitotically inactivated by 2 ug/mL Mitomycin C, as previously described [33]. In parallel with nonadherent cells harvested from day 12 of hematopoietic differentiation, human UCB specimens were obtained from Ramathibodi Hospital (COA. MURA2023/305) and National Blood Centre, Thai Red Cross Society (COA No. NBC6/2022) and collected the UCB-derived mononuclear cells using Lymphoprep™ density gradient medium (STEMCELL Technologies). Afterward, both cell types were magnetically sorted using EasySep™ Human CD34 Positive Selection Kit II (STEMCELL Technologies) to isolate UCB- and hiPSC-derived CD34^+^ cells. For LDA, hiPSC-derived cells at determined cell doses (10,000/5,000/2,500/1,250/625) were then placed in 12 replicates of AFT024-prepared wells per dose with 100 μL LTC medium (Myetocult™ H5100, STEMCELL Technologies) containing 1 μM hydrocortisone (Sigma) and 1% P/S under 5% O_2_ and 5% CO_2_ at 37°C with 95% humidified condition. Fresh LTC medium was half-changed weekly. After five weeks of every single LTC well, co-cultured cells were harvested and performed in the human colony-forming unit (CFU) assay for another two weeks, and the well with/without forming colonies were scored to calculate the frequency of stem cells using L-Calc™ software (STEMCELL Technologies). In the same process, LDA was utilized to test UCB-derived cells as a positive control, but a half-lower density of cell doses (5,000/2,500/1,250/625/312.5) per well.

### MethoCult™ CFU assay of LTC-IC and fresh hiPSC-derived HSPCs with cell morphology

Whole cells from the LTC, as well as nonadherent (5 × 10^4^) cells from hematopoietic differentiation, were harvested and resuspended in Iscove-modified Dulbecco medium (Hyclone) and mixed with a methylcellulose-based medium (MethoCult™ H4636, STEMCELL Technologies). The colonies were scored according to standard morphological criteria after 14 days of incubation at 37 ºC, 5% CO_2_, and 95% humidity. Furthermore, colonies from hiPSC-derived nonadherent cells were individually collected for May Grunwald-Giemsa staining (Additional file 1: supplemental experiment procedures) and microscopic observation.

### RNA preparation

Human UCB specimens were obtained and isolated to UCB-derived CD34^+^ cells as described above. In parallel, hiPSC-derived CD34^+^CD45^+^ cells were isolated using Fluorescence-activated cell sorting (FACS). Both cell types were harvested for total RNA isolation using the Qiagen RNeasy Plus Micro Kit (Qiagen), following the manufacturer’s protocol (Additional file 1: Fig. S3). The RNA concentration and quality were estimated using a NanoDrop™ 2000 Spectrophotometer (ThermoFisher Scientific).

### NanoString® mRNA analysis

RNA concentration was adjusted to 20 ng/μL each, following the manufacturer’s instructions, for utilization in the nCounter® Stem Cell Panel and Assay (NanoString Technologies), which includes 770 probes (Additional file 1: supplemental experiment procedures). The panel was employed to determine the gene expression patterns specific to stem cell characteristics and properties, enabling the investigation of the underlying mechanisms. This assay was conducted using four biological replicates from magnetic-sorted and FACS samples.

### Quantitative real-time PCR (qRT-PCR) analysis

Total RNA from individual samples was isolated as described above, following the manufacturer’s instructions. RNA was then reverse transcribed by the SuperScript™ III First-Strand Synthesis System (Invitrogen). The expression of mRNA was examined using specific oligonucleotides (listed in Additional file 1: Table S2) and SYBR Green (Bio-Rad) on an ABI PRISM 7500 Sequence Detection System with analytical software (ThermoFisher Scientific). The levels of targeted gene expression were analyzed with ΔΔCT, normalized to the expression of GAPDH, to demonstrate the fold change (FC) relative to the negative control.

### Immunoblotting analysis

Magnetically sorted hiPSC-derived CD34^+^ cells were washed three times with cold PBS, lysed in protein-preservative buffers, and stepwise prepared for polyacrylamide gel electrophoresis, subsequently transferred to membranes by electroblotting as previously described [30]. Afterward, membranes were incubated with the primary antibodies: p-YAP1 (phospho-Ser127, 1:500, Abcam), YAP1 (1:1000, Abcam), p-WWTR1 (phospho-Ser89, 1:500, Invitrogen), WWTR1 (1:1000, Invitrogen), and GAPDH (1:20,000, Invitrogen), overnight at 4 °C. Subsequently, membranes incubated with antibodies were washed five times with Tris-buffered saline plus Tween 20 and incubated with HRP-conjugated secondary antibodies. Targeted signals were exposed with HRP Substrate and detected by Azure 600 (Azure Biosystems). Detailed information on these antibodies and reagents is provided in Additional file 1: Table S1.

### Immunofluorescence analysis

Magnetically sorted hiPSC-derived CD34^+^ cells were washed, single-cell resuspended, and loaded onto cytospin-prepared slides (Additional file 1: supplemental experiment procedures). Slide-attached cells were then fixed with 4% paraformaldehyde for 20 min. Permeabilization was performed with 0.2% Triton X within 10 min and 2% BSA blocking in PBS for 1 h. The cells were incubated with primary antibodies p-TAZ (1:100), TAZ (1:100), p-YAP1 (1:100), and YAP1 (1:500) for 3 h. Then, cells were rinsed with PBS for 5 min three times. Next, cells were incubated with fluorophore-conjugated secondary antibodies (1:500) for 1 h and washed with PBS for 5 min three times. The stained cells on microscope slides were mounted, sealed, and imaged with a confocal laser scanning microscope (Zeiss LSM 900). Detailed information on these antibodies is provided in Additional file 1: Table S1.

### Bioinformatics analysis

Data normalization, visualization, and statistical analysis were performed by nSolver™ software, utilizing the geometric mean of the top twelve housekeeping genes. Significance of differentially expressed genes (DEGs) was considered by the criteria of a *p*-value < 0.05 and FC < -2 or > 2. The heatmap expression plots were generated using nSolver™ visualization and MetaboAnalyst 5.0, accessible at https://www.metaboanalyst.ca. The volcano plots for gene expression were generated using VolcaNoseR, accessible at https://huygens.science.uva.nl/VolcaNoseR/. WEB-based GEne SeT analysis Toolkit (Webgestalt) was employed for gene enrichment analysis, accessible at http://webgestalt.org/. The significant threshold for this analysis was set at false discovery rates (FDR) < 0.5. The data based on Kyoto Encyclopedia of Genes and Genomes (KEGG), available at https://www.genome.jp/kegg/pathway.html, was utilized to map the related pathways.

### Statistical analysis

Unless stated, the data based on a minimum of biological triplicates (*n* ≥ 3) were represented as mean ± SEMs. Significant differences were analyzed using paired student's *t*-test or repeated-measures one-way ANOVA with Dunnett's multiple comparisons, where appropriate, on GraphPad Prism 9 (GraphPad software). The statistical difference at a *p*-value of less than 0.05 was considered significant.

## Results

### ASPP 049 promoted hematopoietic differentiation of hiPSCs into HSPCs

**Fig. 1:**
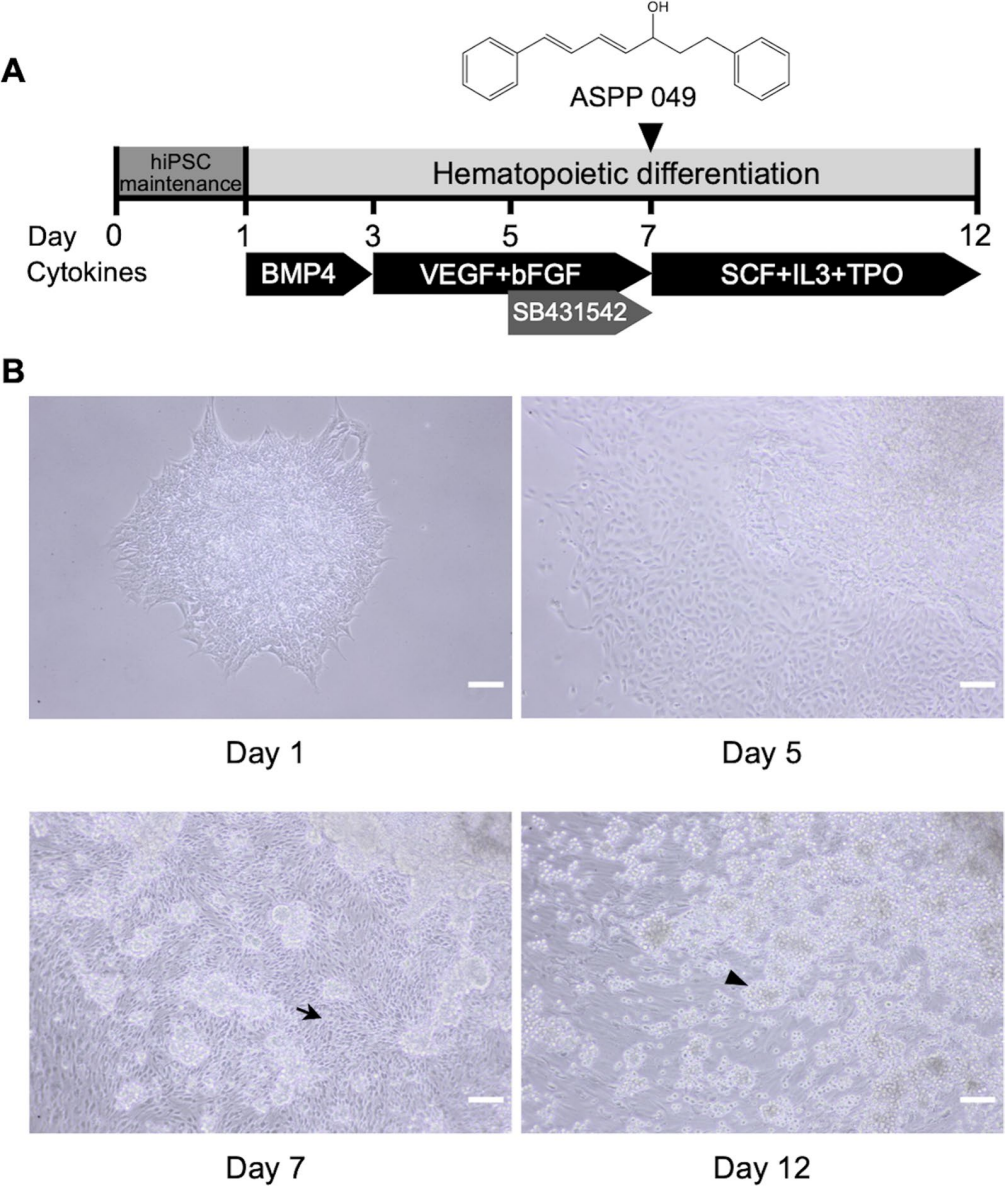
Hematopoietic differentiation for HSC generation from human induced pluripotent stem cells. **A** schematic protocol of hiPSC differentiation into HSPCs with chemically defined factors. Cells were treated with either a diarylheptanoid ASPP 049 or vehicle control (DMSO) starting on day 7 with medium changes. **B** Representative morphologies of differentiating cells in adherent (black arrow) and nonadherent (black arrowhead) fractions during the differentiation: scale bars, 100 μm

To determine the effect of ASPP 049 on the differentiation process, we utilized a serum- and feeder-free directed differentiation protocol, as previously described [19], for generating HSCs in the presence of ASPP 049 (Fig. 1A). The critical step of hematopoietic differentiation is hemato-endothelial induction [10, 14], the checkpoint in facilitating HSC generation in vitro [8, 20, 24, 34]. Thus, we initially treated ASPP 049 on day 7, having found that hiPSCs were morphologically transformed into endothelial-like cells (black arrow), then successfully generating HSC-like cells (black arrowhead) on day 12 (Fig. 1B).

**Fig. 2:**
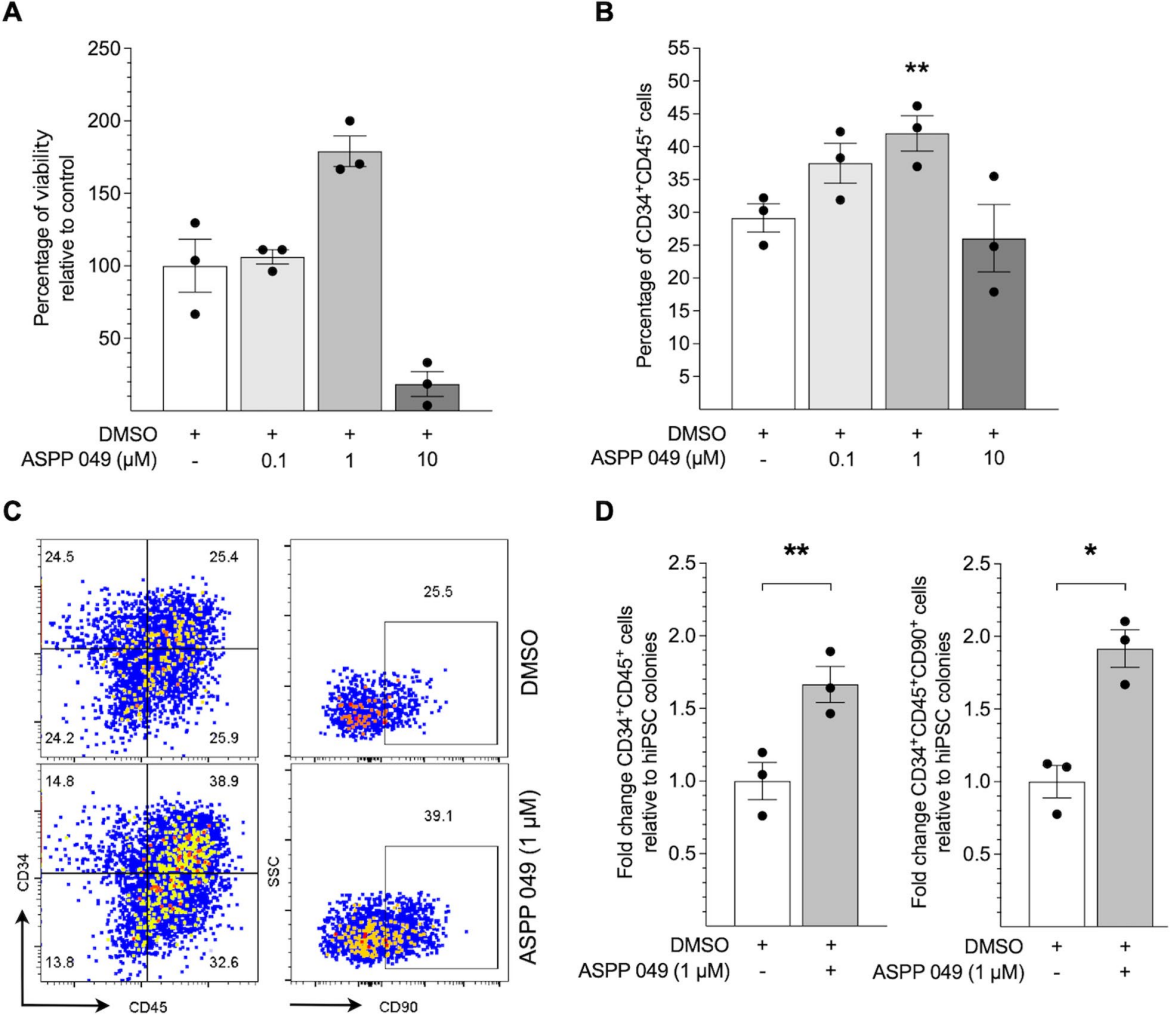
Optimal concentration of ASPP 049 increases hiPSC-derived HSPCs in vitro. **A** Cell viability of hiPSC-derived nonadherent cells is relative to DMSO vehicle control and **B** CD34^+^CD45^+^ fractions represented as the percentage of flow cytometry gating in the presence of various concentrations (0.1, 1, and 10 μM) of ASPP 049. **A** and **B** Individual data are represented on bar charts with mean ± SEM error bars from three biological replicates. **p < 0.01 compared with DMSO (repeated-measures one-way ANOVA with Dunnett's multiple comparisons). **C** Representative flow cytometry analysis of hiPSC-derived nonadherent cells harvested on day 12, assessed by phenotypically defined markers of HSPCs (CD34^+^CD45^+^) and primitive HSPCs (CD34^+^CD45^+^CD90^+^). **D** Fold change analysis of the hematopoietic phenotypes of derived cells relative to hiPSC-seeded colonies in the presence of 1 μM ASPP 049. Individual data relative to DMSO are represented on a bar chart with mean ± SEM error bars from three biological replicates. *p < 0.05, **p < 0.01 compared with DMSO (paired student’s *t*-test)

To find the optimal supplementary condition for ASPP 049, we tested the cytotoxicity of various concentrations on hematopoietic committed cells. We assessed cell viability using a Trypan blue exclusion assay on day 12. Normalizing the results with vehicle control (DMSO, cell viability 100%), we observed an increase in cell viability to 179.01 ± 10.55% at a concentration of 1 µM ASPP 049, while a concentration of 10 µM ASPP 049 decreased cell viability to 18.52 ± 8.55% (Fig. 2A) as well as total nucleated cell number relative to hiPSC-seeded colonies of 1 µM ASPP 049 (8.40 ± 1.14 × 10^3^ cells, *p* = 0.038) and 10 µM ASPP 049, (2.52 ± 1.87 × 10^3^ cells, *p* = 0.154) compared to DMSO (3.94 ± 1.04 × 10^3^ cells) (Additional file 1: Fig. S1A). We also detected a higher efficiency in HSC generation with 1 µM ASPP 049 treatment, as indicated by a significant increase in the percentage of CD34^+^CD45^+^ markers (ASPP 049, 42.03 ± 4.66% vs. DMSO, 29.17 ± 2.15%, *p* = 0.0041, Fig. 2B) and CD34^+^CD45^+^ cell number (ASPP 049, 3.58 ± 0.64 × 10^3^ cells vs. DMSO, 1.12 ± 0.31 × 10^3^ cells, *p* = 0.0360) on day 12 (Additional file 1: Fig. S1B and D) using flow cytometry with the gating strategy (Additional file 1: Fig. S1C). Additionally, ASPP 049 treatment significantly increases the ratios of CD34^+^CD45^+^ HSPCs (ASPP 049, 1.67 ± 0.13-fold vs. DMSO, 1.00 ± 0.13-fold, *p* = 0.0013) as well as CD34^+^CD45^+^CD90^+^ primitive HSPCs [26, 35] (ASPP 049, 1.92 ± 0.13-fold vs. DMSO, 1.00 ± 0.11-fold, *p* = 0.0270) relative to the initial number of hiPSC-seeded colonies (Fig. 2C, D). Thus, ASPP 049 induced the proliferation of HSPCs generated from hiPSCs.

### ASPP 049 escalated various progenitors during EHT

**Fig. 3:**
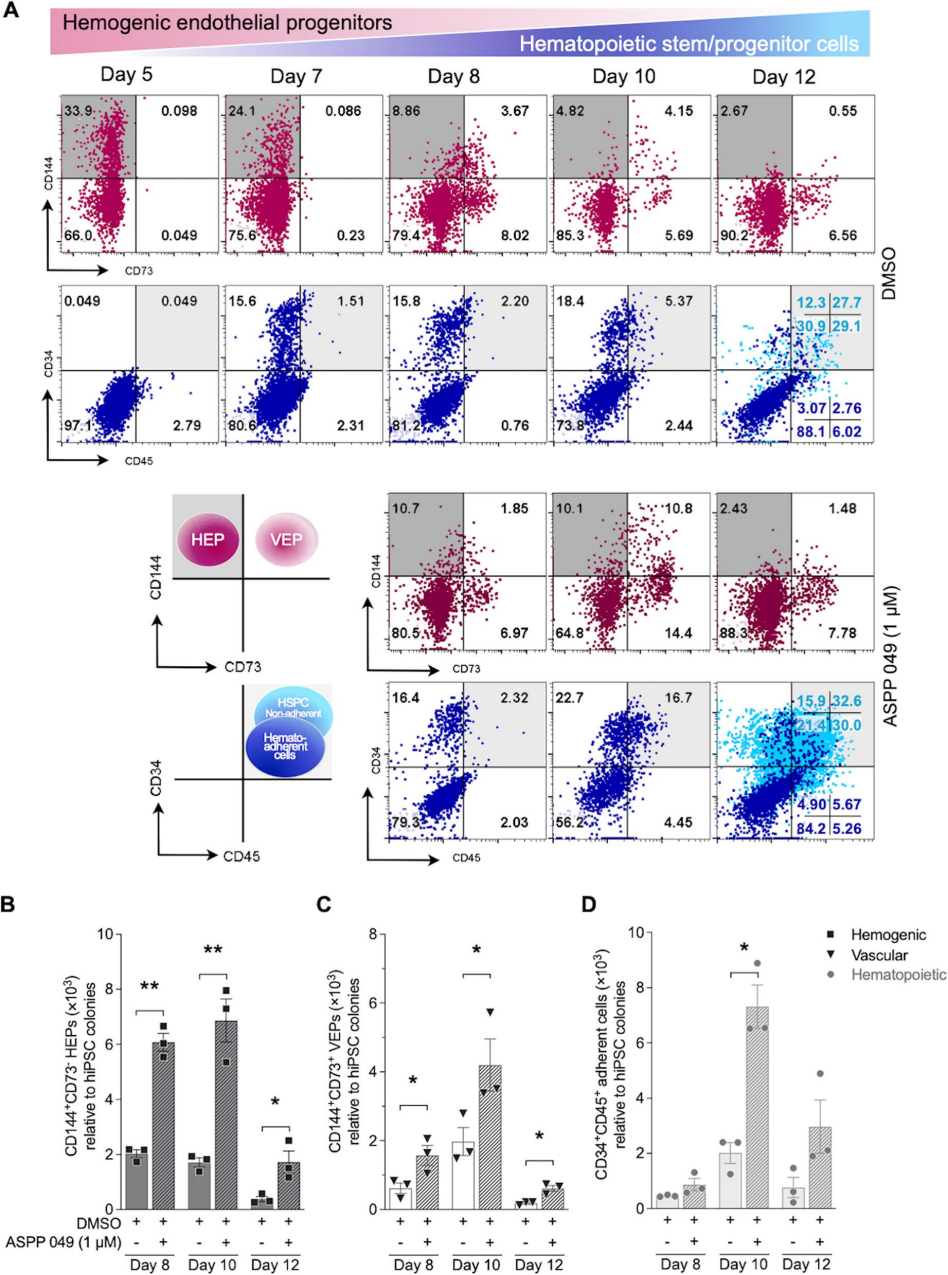
ASPP 049 modulates the developmental endothelial progenitors through endothelial-to-hematopoietic transition (EHT). **A** Kinetics of flow cytometry analysis of ASPP 049 (1 μM) on EHT defined by the expression of endothelial progenitor markers (CD144 and CD73) and hematopoietic markers (CD34 and CD45). The developmental endothelial potency of adherent cells: hemogenic endothelial progenitors (HEP, CD144^+^CD73^−^), vascular endothelial progenitors (VEPs, CD144^+^CD73^+^), and hematopoietic committed cells (CD34^+^CD45^+^) during hematopoietic differentiation are shown. **B–D** Statistical analysis of each endothelial progenitor number of different time points relative to hiPSC-seeded colonies. Individual data are represented on a bar chart with mean ± SEM error bars from three biological replicates. *p < 0.05, **p < 0.01 compared with DMSO vehicle control simultaneously (paired student's *t*-test)

During hematopoietic development or ex vivo differentiation, hemato-endothelial origin cells with hemogenic and angiogenic characteristics, characterized by surface protein markers [10], play an essential role in HSC specification and emergence [12, 13]. To evaluate the effects of ASPP 049 on distinguishing cell types, we assessed the endothelial phenotypes of differentiating adherent cells derived from hiPSCs. As shown in Fig. 3A, adherent cells from days 5 to 12 during the differentiation, both with and without ASPP 049, were characterized to determine endothelial markers that discriminate between hemogenic endothelial progenitors (HEPs, CD144^+^CD73^−^) and vascular endothelial progenitors (VEPs, CD144^+^CD73^+^) [10, 34] through EHT.

On day 7 of the differentiation before ASPP 049 treatment, we observed the markers of VEPs (0.03 ± 0.05%) that modestly increased until day 10, in contrast to HEPs (25.3 ± 1.70%) that slightly decreased until day 12, alongside the expression of CD34 on day 7 and CD45 from day 8 onwards, indicating hematopoietic differentiation. Although CD144 expression declined over time, resulting in a loss of endothelial characteristics by day 12, cells treated with ASPP 049 and vehicle control continued to exhibit HEP markers (ASPP 049, 13.0 ± 2.72% vs. DMSO, 9.23 ± 0.51%) and VEP markers (ASPP 049, 3.46 ± 1.54% vs. DMSO, 2.83 ± 1.23%) on day 8. These markers were highly elevated in ASPP 049-treated HEPs (ASPP 049, 13.5 ± 3.68% vs. DMSO, 5.56 ± 0.64%) and ASPP 049-treated VEPs (ASPP 049, 8.19 ± 2.54% vs. DMSO, 6.99 ± 4.35%) on day 10, accompanied by an increase in the CD34^+^CD45^+^ population. The concurrent upregulation of CD34 and CD45, along with the loss of CD144 on day 12 (~ 2.5% with or without ASPP 049), indicates successful EHT that potentially generates hiPSC-derived CD34^+^CD45^+^ HSPCs (Fig. 3A). Further analysis with flow cytometry and trypan blue exclusion assay revealed a significant three-fold increase in the cell number of HEPs treated with ASPP 049 compared to the initial number of hiPSC-seeded colonies (Day 8: ASPP 049, 6.08 ± 0.33 × 10^3^ HEPs vs. DMSO, 2.03 ± 0.15 × 10^3^ HEPs, *p* = 0.0035; Day 10: ASPP 049, 6.87 ± 0.78 × 10^3^ HEPs vs. DMSO, 1.71 ± 0.17 × 10^3^ HEPs, *p* = 0.0073; Day 12: ASPP 049, 6.87 ± 0.78 × 10^3^ HEPs vs. DMSO, 0.38 ± 0.10 × 10^3^ HEPs, *p* = 0.0480) (Fig. 3B), along with a similar increase in VEPs (Day 8: ASPP 049, 1.57 ± 0.29 × 10^3^ VEPs vs. DMSO, 0.62 ± 0.15 × 10^3^ VEPs, *p* = 0.0476; Day 10: ASPP 049, 4.20 ± 0.76 × 10^3^ VEPs vs. DMSO, 1.97 ± 0.41 × 10^3^ VEPs, *p* = 0.0131; Day 12: ASPP 049, 0.61 ± 0.09 × 10^3^ VEPs vs. DMSO, 0.18 ± 0.03 × 10^3^ VEPs, *p* = 0.0247) (Fig. 3C). Accordingly, the normalized cell number of CD34^+^CD45^+^ adherent cells (hematopoietic committed cells) under ASPP 049-supplemented conditions significantly increased up to four-fold on day 10 (ASPP 049, 7.31 ± 0.78 × 10^3^ cells vs. DMSO, 2.01 ± 0.38 × 10^3^ cells, *p* = 0.0226) and up to three-fold on day 12 (Fig. 3D). These findings on the differentiating progenitors indicated the spontaneously gradual acquisition with time of hematopoietic committed characteristics instead of endothelial characteristics. In addition, ASPP 049 supplementation maintained the existence of HEPs and VEPs, facilitating HSPC generation. Taken together, ASPP 049 not only exerted a proliferative effect on late HSPCs but also enhanced the expression of hemato-endothelial origins and early hematopoietic committed cells without interrupting EHT in vitro.

### ASPP 049 improved self-renewal and multi-lineage differentiation with unbiased hematopoietic progenitor lineage ratio in vitro

**Fig. 4:**
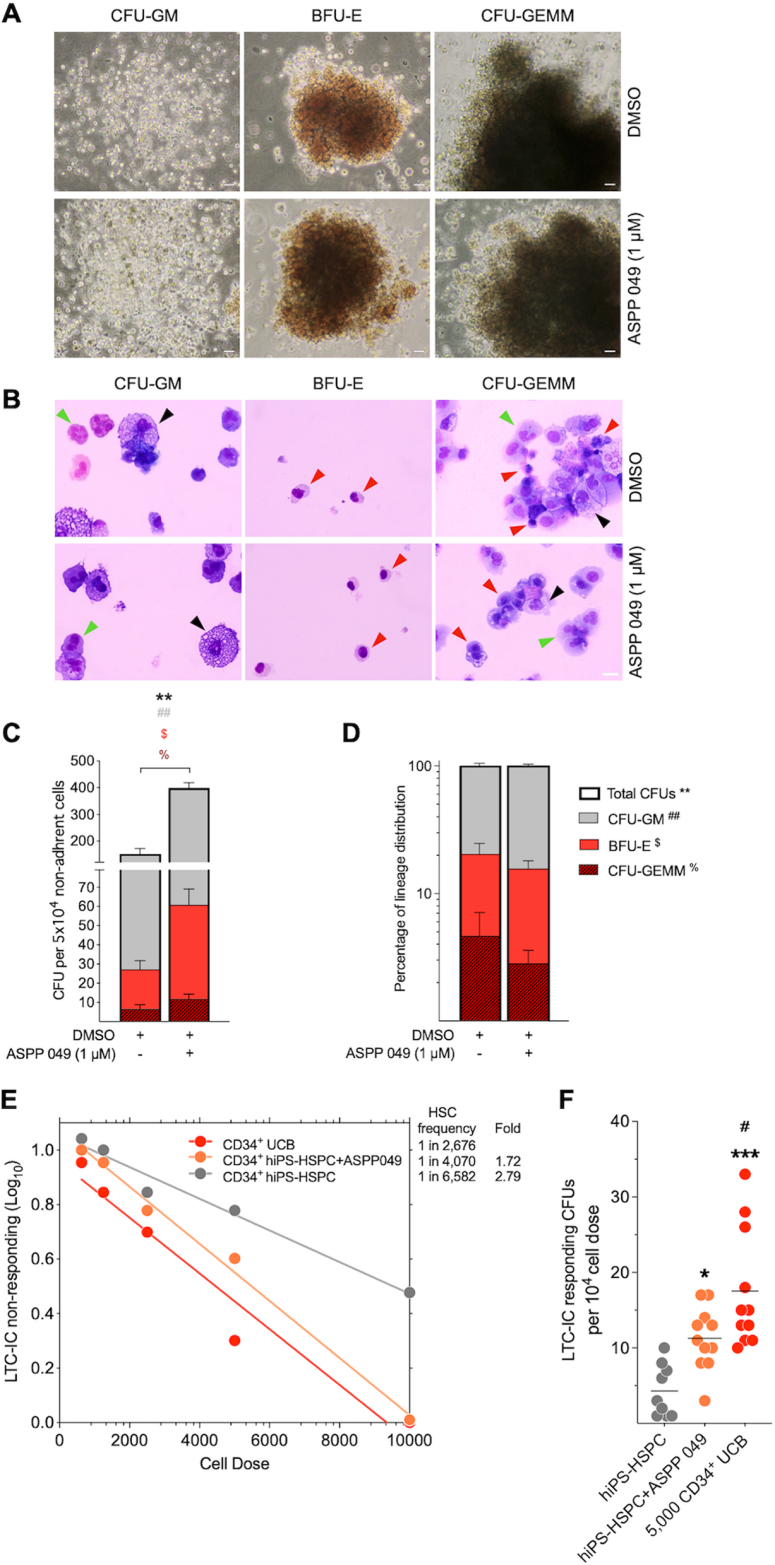
ASPP 049-treated cells promote self-renewal and multi-lineage differentiation potential with unbiased lineage distribution. **A** The blood cell colony-forming potentials of nonadherent (5 × 10^4^) cells from day 12 differentiation were harvested into single-cell suspension and cultured in Methocult™ medium for 2 weeks, evaluating the multi-lineage differentiation potentials. Representative morphologies of blood cell colony: CFU-GM (left), BFU-E (middle), CFU-GEMM (right). Scale bars, 50 μm. **B** May Grunwald-Giemsa staining of CFU-GM, BFU-E, and CFU-GEMM from harvested colonies. Green arrowhead: granulocytic neutrophils, black arrowhead: macrophages, red arrowhead: orthochromatic erythrocytes. Scale bars, 100 μm. **C** Statistical analysis of colony formation was classified and scored under a bright-field microscope according to color, colony size, and cellular compositions: CFU-GM (grey), BFU-E (red), CFU-GEMM (dark red), and total CFUs. **D** Blood lineage distribution represents the ratio of all CFUs between nonadherent cells harvested from ASPP 049 (1 μM)- and DMSO-supplemented conditions. Data are represented as mean ± SEM from three biological replicates. Remarks: * represents statistics of total CFUs, # represents statistics of CFU-GM, $ represents statistics of BFU-E, and % represents statistics of CFU-GEMM. **p < 0.01, ^##^*p* < 0.01, ^$^*p* < 0.05, ^%^*p* < 0.05 compared with DMSO vehicle control (paired student's *t*-test). **E** Limiting dilution analysis of long-term culture-initiating cells using different cell doses indicates the effect of ASPP 049 on maintaining a frequency of HSC-like cells after 5 weeks of LTC on AFT024 plus 2 weeks for MethoCult™ clonogenic ability, compared with positive control UCB-derived CD34^+^ cells. **F** At the highest doses of tested cells (hiPSC-derived CD34^+^ (1 × 10^4^) cells and UCB-derived CD34^+^ (5 × 10^3^) cells), frequency of blood colony formation was scored from positively responded LTC among hiPSC-derived HSPCs, hiPSC-derived HSPCs + ASPP 049, and UCB-derived HSPC. Each dot represents the number of clonogenic cells in a single LTC plus MethoCult™ wells with individual data from technical replicates: *n* = 9, *n* = 11, and *n* = 11, respectively. Remarks: * represents statistics compared with hiPSC-derived HSPCs, and # represents statistics compared with hiPSC-derived HSPCs + ASPP 049. *p < 0.01, ***p < 0.01, ^#^*p* < 0.05 (one-way ANOVA with Dunnett’s multiple comparison)

Colony-forming unit (CFU) assay was conducted to evaluate the multi-lineage differentiation potential of hematopoietic progenitors derived from hiPSCs. The cells supplemented with ASPP 049 were able to completely differentiate into CFU-GM (CFU of granulocytes and macrophages, BFU-E (burst-forming unit of erythrocytes), and CFU-GEMM (CFU of granulocytes, erythrocytes, megakaryocytes, and macrophages), similar to the pattern of the vehicle control (Fig. 4A). To assess each cell type within their representative colonies, we manually collected each CFU for cytospin and May Grunwald-Giemsa staining. The results from each colony showed morphologically matched hematopoietic cells themselves, with no observed differences in granulocytes or neutrophils (green arrowhead), macrophages (black arrowhead), or orthochromatic erythrocytes (red arrowhead) between treatment and control groups (Fig. 4B). Further analysis revealed ASPP 049 treatment showed a significant increase in the number of total colonies (ASPP 049, 397.80 ± 18.54 CFU vs. DMSO, 150.00 ± 27.80 CFU, *p* = 0.0053), including CFU-GM (ASPP 049, 337.80 ± 30.12 CFU vs. DMSO, 122.80 ± 34.87 CFU, *p* = 0.0050), BFU-E (ASPP 049, 49.00 ± 12.74 CFU vs. DMSO, 20.67.80 ± 6.28 CFU, *p* = 0.0244), and CFU-GEMM (ASPP 049, 11.83 ± 1.45 CFU vs. DMSO, 6.50 ± 1.16 CFU, *p* = 0.0334) (Fig. 4C). Interestingly, we found that the percentage of lineage distribution based on CFU capacity did not show an increase in any specific lineage from ASPP 049-treated cells (Fig. 4D), indicating unbiased lineages for multipotency of generated HSPCs.

The LTC-IC assay assesses clonogenic cell output from human LTC-initiating cell input (self-renewal cells) over the limiting dilution of cell doses. It is available for the long-term hematopoietic culture system to demonstrate the functionally primitive human HSPCs [33, 36]. Long-term co-culture with a stromal feeder and continuous reconstitution of blood-lineage colonies can determine the frequency of stem cells maintaining repopulating activity in vivo [32, 37]. The LDA revealed that ASPP 049 profoundly affected HSPCs, showing a 1.72-fold reduction in stem cell frequency compared with the UCB-positive control (ASPP 049, with a 95% CI of 1/4981–1/3325 (frequency = 1/4070) vs. UCB, with a 95% CI of 1/3263–1/2194 (frequency = 1/2676), p = 0.0551), whereas HSPCs without the compound showed a 2.79-fold reduction (DMSO, with a 95% CI of 1/8198–1/5285 (frequency = 1/6582) vs. UCB, with a 95% CI of 1/3263–1/2194 (frequency = 1/2676), *p* = 0.00006). This experiment was calculated using Poisson statistics to compare the ratio of proportions (Fig. 4E). CFU assay of the highest cell dose after LTC of ASPP 049-supplemented HSPCs resulted in a higher frequency compared with the negative control (DMSO, 4.33 ± 1.16; *n* = 9 vs. ASPP 049, 11.27 ± 1.251 CFU; *n* = 11, *p* = 0.0192), which is close to but still lower than the UCB-positive control (ASPP 049, 11.27 ± 1.251 CFU; *n* = 11 vs. UCB, 17.55 ± 2.368 CFU; *n* = 11, *p* = 0.0265) (Fig. 4F). Although incomparable to the potential of UCB, it is notable that ASPP 049 explores the improving effects on the self-renewal HSC compartment derived from hiPSCs.

Likewise, the erythroid-differentiating potential of renewing sources, such as hiPSCs, has been attractive in regenerative medicine [38]. Consequently, we investigated this potential using a three-stage erythroid differentiation process (Additional file 1: supplemental experiment procedures and Fig. S2A). Focusing on the accelerated differentiation of the early erythroid stage on D5E, the effect of ASPP 049 did not disturb the erythroid maturation of hiPSC-derived HSPCs in the later days (Additional file 1: Fig. S2B–E). Variations in globin expression were demonstrated by qRT-PCR (Additional file 1: supplemental experiment procedures), revealing dissimilar ratios of ⍺- and β-like globin mRNAs (⍺ + ζ/β + γ + ε) on D7E between ASPP 049-treated and untreated cells (HSPC to erythroid, 0.82 ± 0.02 ratio vs. HSPC^049^ to erythroid, 0.98 ± 0.03 ratio, *p* < 0.0069) (Additional file 1: Fig. S2F). This difference was particularly notable in higher ⍺-globin mRNA (HSPC to erythroid, 89.60 ± 0.31% vs. HSPC^049^ to erythroid, 91.91 ± 0.24%) in contrast to lower ζ-globin mRNA (HSPC to erythroid, 10.40 ± 0.31% vs. HSPC^049^ to erythroid, 8.09 ± 0.24%) in ASPP 049-treated cells. However, the expression of β-globin mRNA slightly increased in ASPP 049-treated cells (HSPC to erythroid, 0.17 ± 0.03% vs. HSPC^049^ to erythroid, 0.20 ± 0.03%) but the majority was restricted to γ-globin (HSPC to erythroid, 74.15 ± 0.14% vs. HSPC^049^ to erythroid, 73.64 ± 1.65%) (Additional file 1: Fig. S2G). In this study, blood lineage-committed cells derived from ASPP 049-treated cells demonstrated a robust multi-lineage differentiation property without lineage bias, as well as the functional ability of embryonic-to-fetal hemoglobin switching. The cells with or without ASPP 049 demonstrated the ability to differentiate into mature human red blood cells from hiPSCs. However, transitioning to adult β-globin in hiPSC-derived erythroid cells remains challenging [39], and achieving stable expression of β-globin is a major complication.

### Treatment with ASPP 049 is associated with the upregulation of the Hippo signaling

To understand the underlying mechanisms of ASPP 049 effects, we profiled the gene expression of hiPSC-derived HSPCs after treatment with ASPP 049 compared to the control. Total RNA from CD34^+^CD45^+^ sorted samples (hiPS-HSPC) was extracted (Additional file 1: Fig. S3) and subsequently used for NanoString® mRNA analysis, which measured the expression of 770 stem cell-related genes. The readout data were normalized and analyzed by nSolver™ software (NanoString Technologies).

**Fig. 5:**
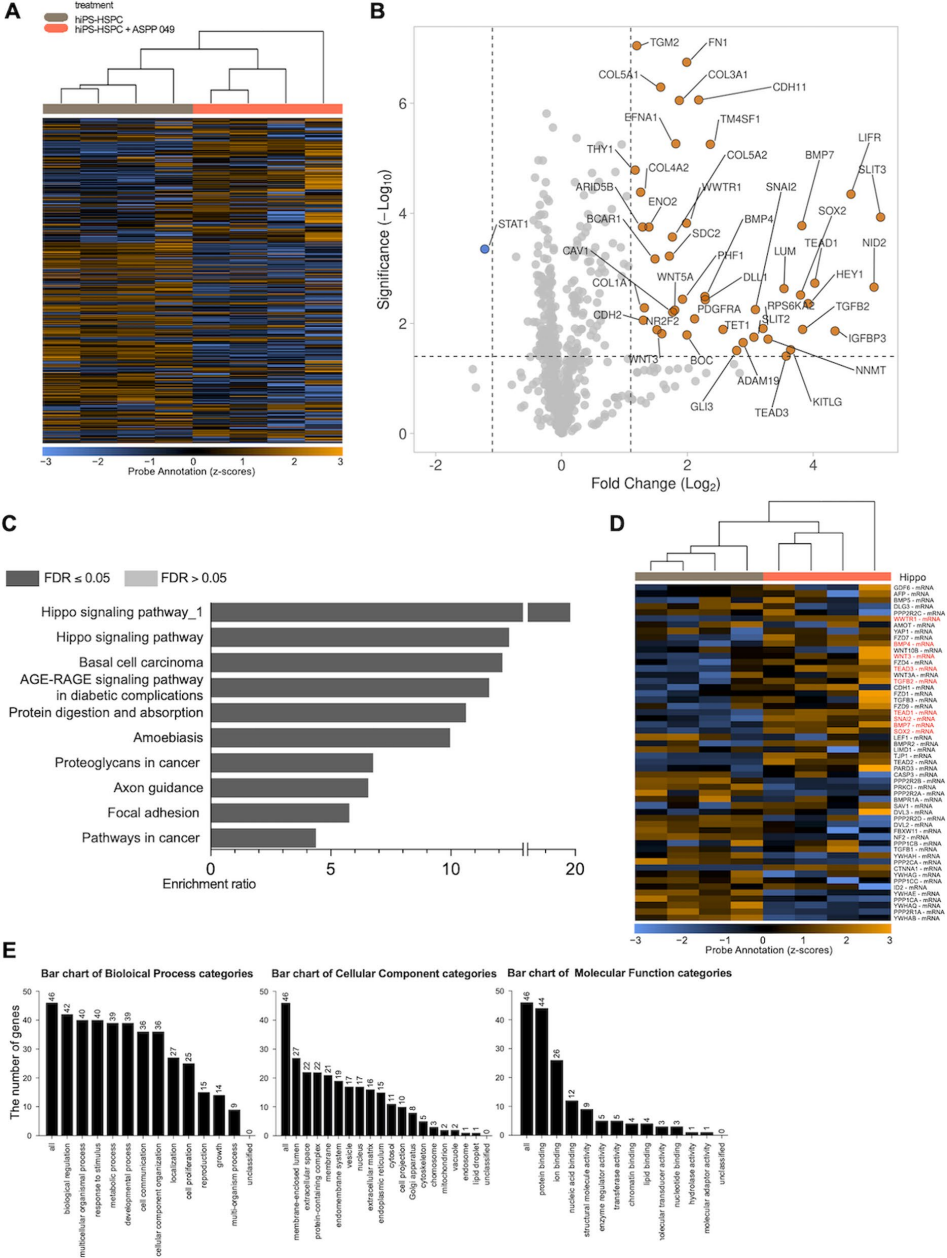
The effect of ASPP 049 on gene expression profiles of the Hippo signaling pathway. **A** Unsupervised heatmap was obtained with sample cluster analysis of gene expression profiles in hiPSC-derived HSPCs (hiPS-HSPC) with or without ASPP 049, *n* = 4. **B** Volcano plot represents the differentially expressed genes (DEGs) between hiPS-HSPC and ASPP 049 treated hiPSC-derived HSPCs (hiPS-HSPC + ASPP 049), mean fold change (log_2_) > 1, *p*-value significance (− log_10_) > 1.4. **C** Gene set enrichment ratio of significant pathways coordinated with transcriptomic alteration in 46 DEGs in hiPS-HSPC and hiPS-HSPC + ASPP 049. **D** Heatmap of the protein-coding genes relates to the Hippo signaling pathway induced in hiPS-HSPC + ASPP 049: no significantly different genes (black label) and significantly different genes (red label). **E** Bar chart of gene set enrichment categorically analyzed in Biological Process, Cellular Component, and Molecular Function. Individual data of expression levels is represented from four biological replicates as a color ranging of mRNA expression levels: blue, downregulated; black, no difference; yellow, upregulated

We aimed to explore the effect of ASPP 049 on global mRNA expression and assessed the transcriptomic alterations in the ASPP 049-treated groups compared to the control. The difference in mRNA profiles between the two groups was observed through an expression heatmap generated using an unsupervised clustering method (Fig. 5A). Additionally, we generated a volcano plot of DEGs among the 770 genes. We found that 45 genes were upregulated and 1 gene was downregulated in the ASPP 049-treated groups compared to controls, with a significant threshold of -log_10_ (*p*-value) > 1.5 and log_2_ (FC) > 1 (Fig. 5B, Additional file 1: Table S3). Overall, the enrichment analysis revealed that the 46 DEGs were associated with multiple pathways, including the Hippo signaling pathway, pathways in cancer, AGE-RAGE signaling pathway in diabetic complications, proteoglycans in cancer, protein digestion and absorption, amoebiasis, axon guidance, basal cell carcinoma, and focal adhesion, respectively. Among these, the Hippo signaling pathway, known to play a role in stem cell biology [40], showed the most significant enrichment ratio based on the lowest FDR score (Fig. 5C, Additional file 1: Table S3). Hippo signaling-related transcriptomic changes of hiPS-HSPC induced by ASPP 049 (hiPS-HSPC + ASPP 049) consistently showed two upregulated gene sets of Hippo signaling pathway_1: WWTR1; TEAD1; TEAD3 and Hippo signaling pathway: WWTR1; TEAD1; TEAD3; SOX2; SNAI2; BMP4; BMP7; TGFB2; WNT3; WNT5A (Fig. 5D, Additional file 1: Table S3). Altogether, this analysis demonstrated that ASPP 049 treatment alters the expression of protein-coding genes involved in stem cell-regulated mechanisms in hiPS-HSPC.

To further identify the biological functions involved in the significant DEGs, we conducted gene set enrichment analysis using Webgestalt. The enriched gene sets in categories of Biological Process, Cellular Component, and Molecular Function reported the effect of ASPP 049 on hiPS-HSPC that mostly correlates to biological regulation, membrane-enclosed lumen, and protein binding, respectively (Fig. 5E).

**Fig. 6:**
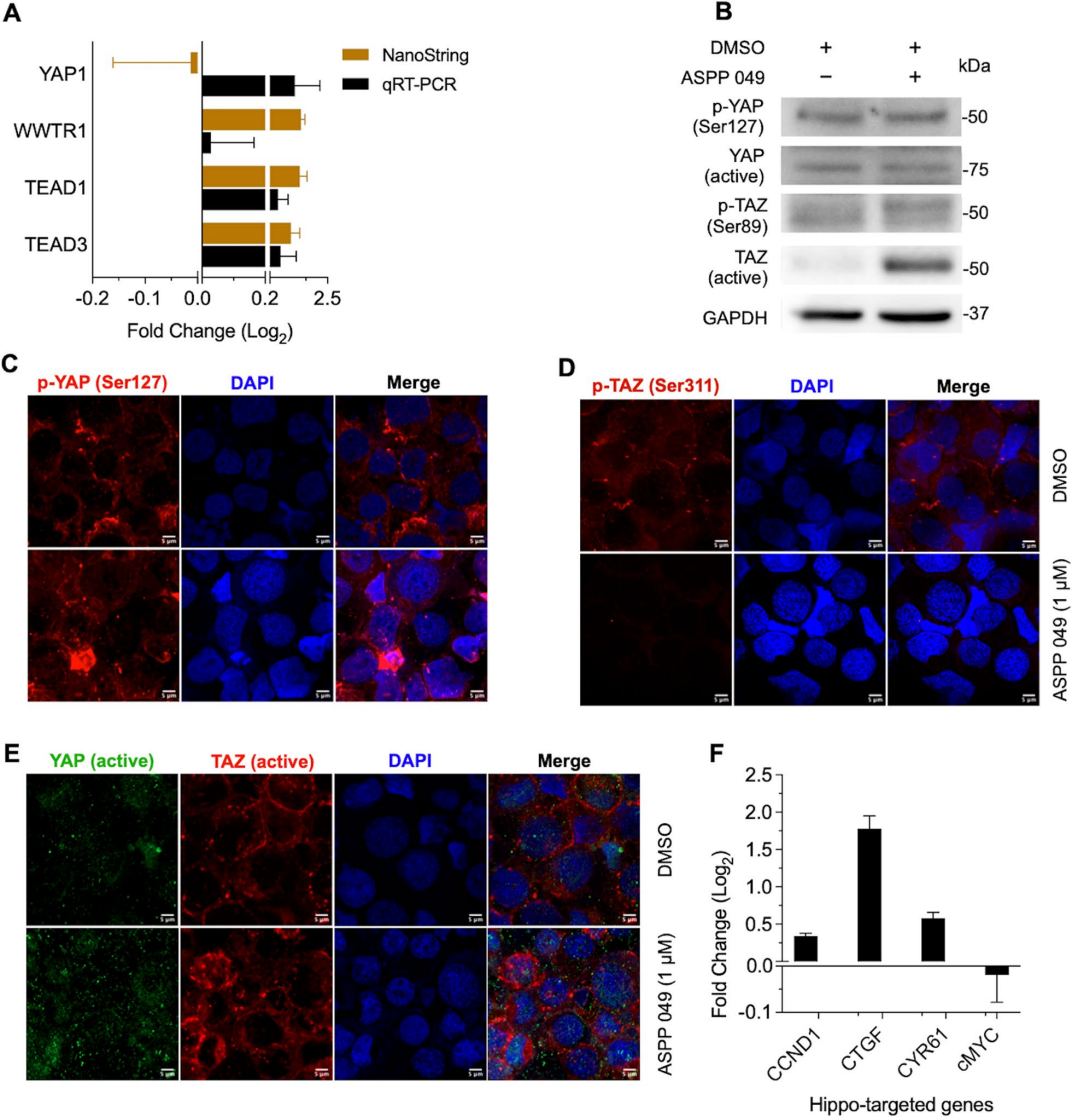
Validation of Hippo signaling-related gene expression profiles in hiPSC-derived HSPCs**. A** Using quantitative real-time PCR compared with NanoString® mRNA analysis, the expression of ASPP 049-associated top significant genes is represented as mean ± SEM of the fold change (log_2_) relative to vehicle control from two biological replicates (each technical triplicates) **B** Immunoblotting analysis of hiPSC-derived CD34^+^ cells on day 12 hematopoietic differentiation with and without ASPP 049 supplementation probed with anti-YAP and anti-TAZ in both active (no phosphorylation) and inactive (phosphorylation) forms. Full-length blots are presented in Additional file 1: Fig. S4. **C** Immunofluorescence analysis of protein expression levels and localizations of these Hippo signals influenced by the effect of ASPP 049 (1 μM) on hiPSC-derived CD34^+^ cells compared to vehicle control from two biological replicates. **D** Quantitative real-time PCR of ASPP 049-associated Hippo-targeted genes represents mean ± SEM of the fold change (log_2_) relative to vehicle control from two biological replicates (each technical triplicate)

Moreover, to validate the expression of Hippo signaling-related genes found in the NanoString® assay, the qRT-PCR assay demonstrated a comparable pattern for those core Hippo signals: WWTR1, TEAD1, TEAD3, and YAP1 (Fig. 6A). Based on protein phosphorylation related to Hippo signaling inactivation, immunoblotting revealed that YAP signals, either phosphorylated Ser127 (p-Ser127) inactive form or non-phosphorylated active form, showed no difference between cells with and without ASPP 049, in contrast to the levels of phosphorylated Ser89 (p-Ser89) inactive form and active form of TAZ. These proteins appeared to play a major role in hiPS-HSPC + ASPP 049, indicating an increase in the active form of TAZ (Fig. 6B, Additional file 1: Fig. S4), consistent with its NanoString® mRNA level. Furthermore, we studied the localization of YAP and TAZ. Immunofluorescence revealed that, as expected, inactive YAP1 (p-Ser127) was mainly localized in the cytoplasm of hiPS-HSPC in both conditions (red, Fig. 6C). Of note, phosphorylated Ser311 (p-Ser311) inactive TAZ was markedly expressed only in the cytoplasm of hiPS-HSPC but not hiPS-HSPC + ASPP 049 in response to the effect of ASPP 049 on hematopoietic cells (red, Fig. 6D). Interestingly, the active forms of YAP and TAZ were localized in the cytoplasmic and nuclear compartments but without the co-localization of YAP-TAZ in the nucleus, indicating their self-governing role in hiPS-HSPCs in both conditions (green and red, Fig. 6E). Particularly, active TAZ in hiPS-HSPC + ASPP 049 was mainly located in the perinuclear site compared with hiPS-HSPC control (red, Fig. 6E).

To confirm the effects of ASPP 049 on Hippo signal-transcriptional activation by either YAP or TAZ, we also demonstrated the expression of Hippo signaling-targeted genes: cMYC, CYR61, CTGF, and CCDN1 and found apparent upregulation except for the cMYC gene (Fig. 6F). Taken together, the higher levels of mRNA and active form proteins of a Hippo signal, WWTR1/TAZ, or even the mRNA of Hippo transcription factors, TEADs influenced by ASPP 049 can improve the hematopoietic derivation and function of HSPCs from hiPSCs.

### The gene expression profiles after ASPP 049 treatment correlate to expression patterns of CD34^+^ cells isolated from umbilical cord blood

**Fig. 7:**
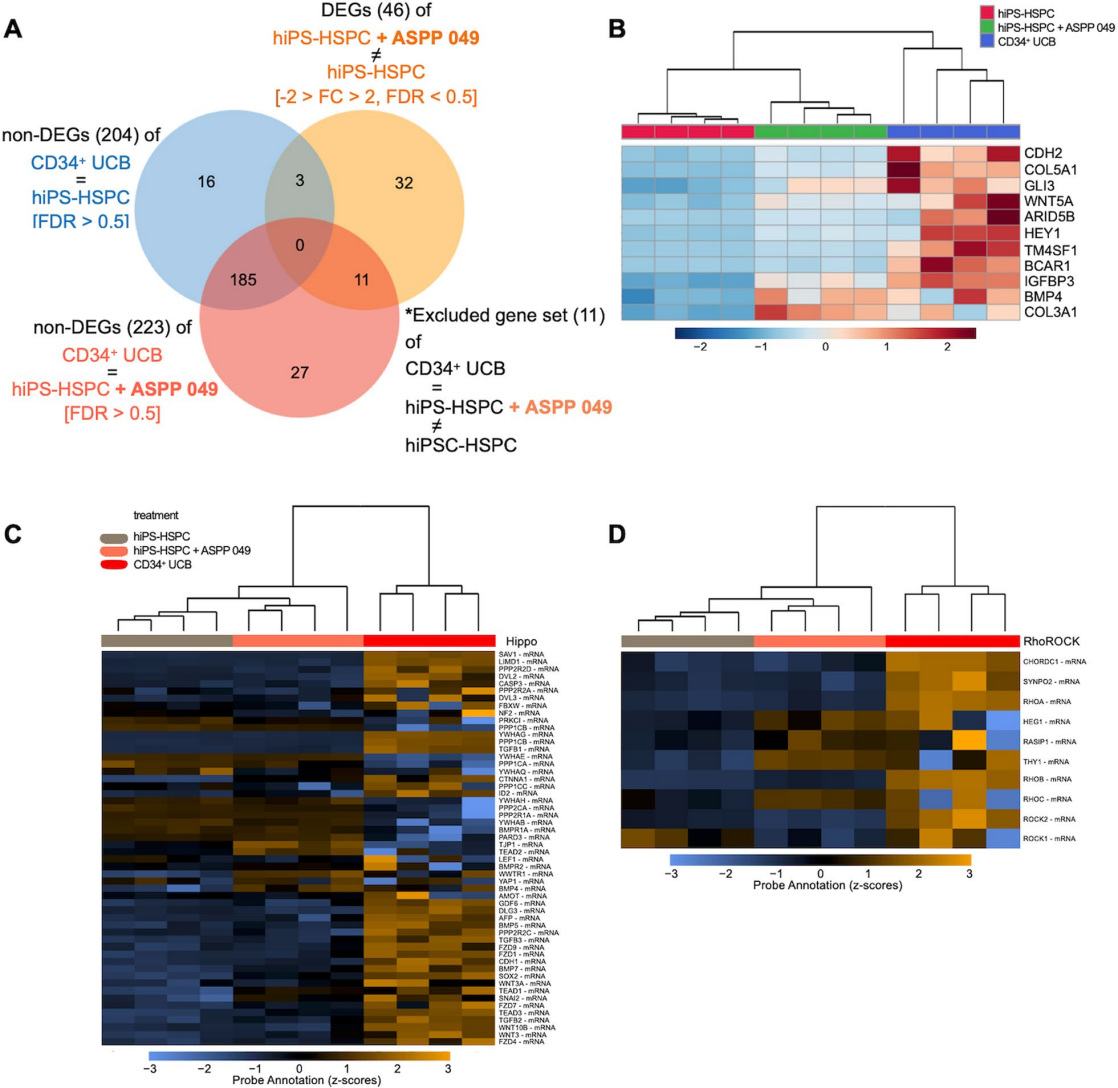
ASPP 049 supplementation induced gene expression patterns correlating with stemness similar to UCB-derived CD34^+^ cells (CD34^+^ UCB). **A** Venn diagram of three different gene lists between key DEGs of hiPS-HSPC vs. hiPS-HSPC + ASPP 049 (gene set 1, orange), non-DEGs of hiPS-HSPC vs. CD34^+^ UCB (gene set 2, blue), and non-DEGs of hiPS-HSPC + ASPP 049 vs. CD34^+^ UCB (gene set 3, red) identified a subset of 11 rescued genes in response to closely correlated patterns of CD34^+^ UCB. **B** Heatmap showing the protein-coding gene expressions of hiPS-HSPC + ASPP 049 (green) were rescued (not upregulated or down-regulated) in similar to CD34^+^ UCB (red) compared with hiPS-HSPC (dark blue). **C** and **D** Heatmap of Hippo signaling-related genes **C** and RhoROCK signaling-related genes **D** showing the induction of hiPS-HSPC + ASPP 049 related to the rescued patterns compared with hiPS-HSPC and CD34^+^ UCB. Individual data of expression levels is represented from four biological replicates as a color ranging of mRNA expression levels: blue, downregulated; black, no difference; yellow, upregulated

In this study, we further investigated the gene expression profiles between UCB-derived CD34^+^ cells (CD34^+^ UCB), which represent clinically relevant HSCs [41], and hiPS-HSPC. The mRNA profiles of the three groups were observed through an expression heatmap (Additional file 1: Fig. S5A). With identifying profile, hiPS-HSPCs might be artificially correlated with CD34^+^ UCB [34]; we used a Venn diagram to compare between three different gene lists. The first comparison was between DEGs of hiPS-HSPC and hiPS-HSPC + ASPP 049 (displayed in orange, Fig. 7A). The second comparison was between non-DEGs of CD34^+^ UCB and hiPS-HSPC + ASPP 049 (displayed in red, Fig. 7A). The third comparison was between non-DEGs of CD34^+^ UCB and hiPS-HSPC (displayed in blue, Fig. 7A). We identified 11 genes of interest from 46 DEGs, including ARID5B, BCAR1, BMP4, CDH2, COL3A1, COL5A1, HEY1, IGFBP3, TM4SF1, WNT5A, and GLI3, by intersecting those non-DEGs, FC < 2 and > -2 with FDR > 0.5 (Fig. 7A). Then, the heatmap of expression pattern was demonstrated in Fig. 7B. Additionally, we performed a transcriptomic comparison using volcano plots, which revealed the fold change and significance of 46 key DEGs after ASPP 049 treatment compared to DEGs involving CD34^+^ UCB (Additional file 1: Fig. S5B, C). From the 11 genes of interest, the transcriptomic changes of hiPS-HSPC induced by ASPP 049 related to BMP4 and WNT5A genes, which are pointed to the Hippo signaling pathway (Fig. 7C, Additional file 1: Table S5). Obviously, the upregulation of genes, including HEG1, RASIP1, RHOC, and particularly THY1 (CD90), correspond to CD34^+^ UCB (Fig. 7D). Interestingly, enrichment analysis indicates that these 11 genes participate in various pathways, especially signaling pathways regulating pluripotency of stem cells. Unexpectedly, they also participate in the Hippo signaling pathway (Additional file 1: Fig. S5D, Table S5), which has been consistently shown to play a role in the effect of ASPP 049 on expanded HSCs, as previously described [29]. The gene sets identified, consisting of 46 DEGs and 11 genes of interest, were analyzed using KEGG for signaling pathway predictions related to the rescue effects of ASPP 049 on mRNA profiles (Additional file 1: Fig. S6A, B).

## Discussion

Hematopoiesis is initiated by HSCs, which regulate all blood cell differentiation beginning during embryogenesis and continue to maintain a constant supply of mature blood cells throughout human life [42]. The clinical application focuses on increasing the number while preserving the stemness properties of HSCs throughout human life. Among heterogeneous progenies via multi-lineage differentiation, self-renewing HSCs are the exclusive characteristics that can be detected by LTC-IC/LDA assay. However, an increased progenitor ability leads to the restriction of self-renewing HSCs [43]. In a recent study, the diarylheptanoid ASPP 049 has been identified as an efficacious small molecule for adult HSCs with a well-defined mechanism of action while maintaining the balance of self-renewal and multipotency [29]. Given this efficiency, we investigated ASPP 049 for its potential in clinical applications for hematological regenerative medicine. Despite recent advancements in the generation of human HSCs through directed conversion [5, 6, 19], there is still a need for a more efficient and accessible strategy with great clinical potential. In this context, a two-dimensional serum- and feeder-free culturing system is highly practical for an efficient HSC generation [44–47].

Additionally, chemically defined small molecules and cytokines have been essential in developing a competent protocol for HSC generation from hiPSCs. Several studies show that cells resembling primary sources prospectively exhibit high functionality in vivo [4, 48]. In this study, we investigated the effect of treating hiPSCs with ASPP 049, including its impact on cell viability, self-renewal, multi-lineage differentiation, and mechanisms of action. A potent small molecule, UM171, known for HSC maintenance and expansion, has been shown to induce hematopoietic derivation in vitro [49]. However, the exact mechanism through which UM171 elicits its response still needs to be determined. Interestingly, hematopoietic cells derived from differentiation systems supplemented with UM171 promote the formation of large and numerous CFU-GM and CFU-GEMM but retain erythroid lineage potential on BFU-E in the methylcellulose-induced differentiation assay [20]. UM171 has been additionally employed for HSC expansion and lymphoid culture of hPSC-derived CD43^+^ cells. This report suggests the potential for blood production through CD34^+^CD45^+^CD41^lo^ expanded cells, enriched granulocytic progenitors, and CD34^+^CD45^+^CD7^+^ lymphoid progenitors specific to NK potential [25]. We observed that ASPP 049, an optional small molecule and ex vivo enhancer of HSC expansion, does not selectively amplify all blood cell colonies when used solely during hematopoietic differentiation from hiPSCs. Consistent with the higher number and presence of CD34^+^CD45^+^CD90^+^ HSC-like markers in primitive HSPCs via ASPP 049 supplementation, LTC-IC assay with LDA indicated an increase in HSC frequency in parallel to the high differentiation potential with the normal distribution of blood lineages, suggesting a proportional proliferation of HSCs within a heterogenous culture population. These findings indicate that establishing culture systems for hiPSC-derived HSPCs can be achieved by utilizing the diarylheptanoid ASPP 049.

Although our in vitro experiments revealed that ASPP 049 influences cell proliferation, self-renewal maintenance, and stable differentiation, the mechanism of action still needs to be identified. Determining the biological pathways involved in the effects of ASPP 049 on hiPSC-derived CD34^+^CD45^+^ HSPCs, Webgestalt and KEGG analysis of NanoString® mRNA profiles revealed that the Hippo signaling was a significantly enriched pathway, consistent with previous findings on mobilized peripheral blood HSCs [29]. From the validation, we found a similar pattern of Hippo-related mRNA expression using different techniques and the highly active TAZ in hiPS-HSPCs supplemented with ASPP 049, indicating the higher activation of Hippo signaling by ASPP 049. Indeed, our previous report indicated that ASPP 049 induces Wnt/β-catenin signaling, resulting in bone cell differentiation [50]. During the endothelial-to-hematopoietic transition, hemogenic endothelial cells with the definitive hematopoietic fate emerge the HSPCs by developing microenvironment niches that regulate Wnt signaling activation [51, 52] and involve with various developmental factors, including YAP or TAZ, in regulating self-renewal and stem cell fate [53]. ASPP 049 may likely act on hiPSCs-derived HSPCs via the temporal connection between these signaling pathways. Synergistic regulations may occur between Hippo and Wnt signals or even so BMP signals revealed by our mRNA profiles of a Hippo signaling pathway-related gene set include WWTR1, TEAD1, TEAD3, SNAI2, SOX2, WNT3, WNT5A, BMP4, BMP7, and TGFB2. Coactivated by TAZ, YAP1 is widely expressed in various stem cells, including hiPSCs and HSPCs [40, 54]. Mechanistically, dephosphorylation of YAP and TAZ leads to their accumulation in the nucleus, where they bind to TEAD1-4 and induce the transcription that promotes proliferation and survival [55]. Several studies have highlighted the impact of YAP/TAZ on human hematopoiesis, particularly the dispensable YAP in the high formation of mesodermal cells toward HSPCs from hiPSCs. However, they are essential in producing lymphocytes, megakaryocytes, and erythrocytes [56, 57]. Our data from ASPP 049-treated CD34^+^CD45^+^ HSPCs show that the most significant DEGs related to the Hippo signaling pathway_1 include TEAD1, TEAD3, and WWTR1 (TAZ). We validated similar mRNA levels using qRT-PCR and observed signal activation through the raised levels of perinuclear-localized TAZ protein. Despite cytoplasm-retaining TAZ (p-Ser89) being spontaneously elevated due to feedback activation [58], degradation-priming TAZ (p-Ser311) was decreased in ASPP 049-treated HSPCs. A report explores YAP/TEADs as a stage-specific regulator that promotes early hematopoietic specification of mouse embryonic hemangioblasts onto HEP, and it is nuclear localized before the EHT. However, nuclear YAP is absent in hematopoietic committed cells [59]. Our findings regarding the nuclear localization of TAZ, predominantly influenced by ASPP 049, could shed light on its novel characteristics in primitive HSPCs. Similar to findings in murine HSCs, TAZ alone potentially plays a role in protecting aging characteristics in old HSCs [60]. To verify signal activation, we transcriptionally tested the expression levels of Hippo-targeted genes, including CTGF, CCDN1, cMYC, and CYR61 (TGF-β-induced factor, as same as NanoString® SNAI2 (Slug), which plays a role in maintaining stemness [61]), which showed upregulation in all genes except cMYC, with no changes as expected from NanoString® mRNA profiling (Additional file 1: Fig S6A). We also noticed the elevation of SOX2 pluripotency-related mRNA levels (Additional file 1: Fig S6A, Table S3), which has been reported to be regulated by the crosstalk of TAZ onto NANOG, a master regulator of stemness [62]. Thus, the TAZ co-activator could be independent of YAP in inducing self-governing activity. Consistent with the higher fold change of CD34^+^CD45^+^CD90^+^ primitive HSPCs, THY1 mRNA (CD90), among 46 DEGs, showed a significant increase of up to two-fold in ASPP 049-treated cells. These findings suggest that the TAZ/TEAD-mediated Hippo pathway, whether co-activated with YAP, might contribute to the HSC fate and stemness of the cells derived from hiPSCs after ASPP 049 supplementation.

To examine the mRNA profiling of ASPP 049-treated cells close to CD34^+^ UCB, we performed a comparative analysis of three sets of DEGs using the same criteria. Our findings revealed that 11 genes were identified as key DEGs (hiPS-HSPC + ASPP 049 vs. hiPS-HSPC) that were not significantly expressed in hiPS-HSPC + ASPP 049 and were excluded by two of the non-DEG groups (CD34^+^ UCB vs. hiPS-HSPC and CD34^+^ UCB vs. hiPS-HSPC + ASPP 049). These 11 genes included ARID5B, BCAR1, BMP4, CDH2, COL3A1, COL5A1, HEY1, IGFBP3, TM4SF1, WNT5A, and GLI3, which were further analyzed using Webgestalt plus KEGG analysis. Our re-analysis revealed that out of the key DEGs, 46 genes displayed individual changes. Notably, among the 11 genes retrieved from this analysis, we observed a correlation between the Hippo signaling pathway and signaling pathways regulating pluripotency of stem cells in response to BMP4/WNT5A-mediated ligands, as demonstrated by the enrichment ratio. It suggests that ASPP 049 activated the Hippo signaling in hiPS-HSPCs similar to CD34^+^ UCB.

## Conclusion

This study indicates that the supplementary use of diarylheptanoid ASPP 049 significantly enhances the hematopoietic differentiation potentials of hiPSCs, particularly during the hemogenic/vascular endothelial stages. An increase in the relative cell number and improved hematopoietic qualities are promoted by ASPP 049 through the expansion of self-renewal HSCs without any observable bias toward specific lineages, as evidenced by the similarity in blood cell colony size and morphology. ASPP 049 also shows no disturbed effect on the functional erythroid differentiation. At the molecular level, ASPP 049 exerts its effects on hiPSC-derived HSPCs through a mechanism similar to the HSC expansion system, specifically the Hippo signaling pathway. By uncovering the pathway, we have identified the potential molecule WWTR1/TAZ that could serve as a promising target for hematological therapies to introduce more effective strategies for in vitro hematopoietic differentiation. However, further experiments are required to understand the comprehensive in vivo engraftment capability of ASPP 049-activated hiPSC-derived HSPCs. Our findings shed light on the action of ASPP 049 on HSC differentiation from hiPSCs and provide a scientific rationale for using this small molecule to generate HSPCs for hematological disorder treatments.

### Supplementary Information

**Additional file 1.** Supplementary figures and tables.

## Data Availability

All data in this study are included within the published article and additional file. Bioinformatic datasets used and analyzed during the current study are available from the corresponding author upon reasonable request.

## Abbreviations

HSCs: Hematopoietic stem cells
hiPSCs: Human induced pluripotent stem cells
HSPCs: Hematopoietic stem and progenitor cells
LTC: Long-term culture
LTC-initiating cells: LTC-IC
CFU: Colony-forming unit
qRT-PCR: Quantitative real-time polymerase chain reaction
CD: Cluster of differentiation
KEGG: Kyoto encyclopedia of genes and genomes
WWTR1: WW domain containing transcription regulator 1
TAZ: Transcriptional co-activator with PDZ binding motif
UCB: Umbilical cord blood
hESCs: Human embryonic stem cells
HEPs: Hemogenic endothelial progenitors
EHT: Endothelial-to-hematopoietic transition
NK: Natural killer
CD41^lo^: CD41^low^
E8: Essential 8
P/S: Penicillin/Streptomycin
O_2_: Oxygen
CO_2_: Carbon dioxide
°C: Celsius
mM: Millimolar
EDTA: Ethylene diamine tetraacetic acid
min: Minutes
ROCKi: Rho-associated coiled-coil-containing protein kinase inhibitor
LDA: Limiting dilution analysis
DMEM: Dulbecco’s modified eagle medium
FBS: Fetal bovine serum
μM: Micromolar
2-ME: β-Mercaptoethanol
ng/mL: Nanogram per milliliter
BMP: Bone morphogenetic protein
ng: Nanogram
mL: Milliliter
VEGF: Vascular endothelial growth factor
bFGF: Basic fibroblast growth factor
SCF: Stem cell factor
IL-3: Interleukin-3
TPO: Thrombopoietin
DMSO: Dimethyl sulfoxide
PBE: PBS + BSA + EDTA
PBS: Phosphate-buffered saline
BSA: Bovine serum albumin
PerCP: Peridinin–chlorophyll–protein
FITC: Fluorescein isothiocyanate
APC: Allophycocyanin
PE: Phycoerythrin
μg/mL: Microgram per milliliter
RNA: Ribonucleic acid
FACS: Fluorescence-activated cell sorting
GAPDH: Glyceraldehyde 3-phosphate dehydrogenase
FC: Fold change
YAP1: Yes associated transcriptional regulator 1
p-YAP1: Phosphorylated YAP1
Ser: Serine
p-WWTR1: Phosphorylated WWTR1
HRP: Horseradish peroxidase
p-TAZ: Phosphorylated TAZ
DEGs: Differentially expressed genes
Webgestalt: WEB-based GEne SeT analysis Toolkit
FDR: False discovery rates
SEM: Standard error of the mean
ANOVA: Analysis of variance
Fig: Figure
VEPs: Vascular endothelial progenitors
~: Approximately
CFU-GM: CFU of granulocytes and macrophages
BFU-E: Burst-forming unit of erythrocytes
CFU-GEMM: CFU of granulocytes, erythrocytes, megakaryocytes, and macrophages
CI: Confidential interval
D5E: Day 5 erythroid
*⍺*: Alpha
*ζ*: Zeta
*β*: Beta
*γ*: Gamma
*ε*: Epsilon
D7E: Day 7 erythroid
mRNA: Messenger ribonucleic acid
HSPC^049^: ASPP 049-treated HSPC
hiPS-HSPC: CD34^+^CD45^+^ sorted samples
log: Logarithm
AGE: Advanced glycation end products
RAGE: Receptor for AGEs
hiPS-HSPC + ASPP 049: hiPS-HSPC induced by ASPP 049
TEAD: TEA domain transcription factor 1
SOX2: SRY-box transcription factor 2
SNAI2 or Slug: Snail family transcriptional repressor 2
TGFB2: Transforming growth factor beta 2
WNT: Wnt family member
CYR61: Cysteine-rich angiogenic inducer 61
CTGF: Connective tissue growth factor
CCDN1: Cyclin D1
CD34^+ ^ UCB: UCB-derived CD34^+^ cells
ARID5B: AT-rich interaction domain 5B
BCAR1: Breast cancer anti-estrogen resistance protein 1
CDH2: Cadherin-2 or N-cadherin
COL3A1: Collagen type III alpha 1 chain
COL5A1: Collagen type V alpha 1 chain
HEY1: Hes related family bHLH transcription factor with YRPW motif 1
IGFBP3: Insulin like growth factor binding protein 3
TM4SF1: Transmembrane 4 L six family member 1
GLI3: Gli family zinc finger 3
HEG1: Heart development protein with EGF like domain 1
RASIP1: Ras interacting protein 1
RHOC: Ras homolog family member C
THY1 (CD90): Thy-1 cell surface glycoprotein
NANOG: Nanog homeobox
