# Supplementary material for: Improving hematopoietic differentiation from human induced pluripotent stem cells by the modulation of Hippo signaling with a diarylheptanoid derivative
Source: Stem Cell Res Ther. 2024 Mar 3;15:60. doi: 10.1186/s13287-024-03686-4 (PMC10910864; doi:10.1186/s13287-024-03686-4)
Supplement: Supplementary file 1 — Additional file 1. Supplementary figures and tables. [file 13287_2024_3686_MOESM1_ESM.docx]

# **Additional information**

# **Improving hematopoietic differentiation from human induced pluripotent stem cells by the modulation of Hippo signaling with a diarylheptanoid derivative**

Umnuaychoke Thongsa-ad^1^, Anongnat Wongpan^1^, Wasinee Wongkummool^2,3^, Phaewa Chaiwijit^1^, Kwanchanok Uppakara^4^, Gorawin Chaiyakitpattana^1^, Passanan Singpant^2^, Pirut Tong-ngam^2^, Amnat Chukhan^5^, Wachirachai Pabuprappap^6^, Sirapope Wongniam^7^, Apichart Suksamrarn^6^, Suradej Hongeng^8^, Usanarat Anurathapan^8^, Kasem Kulkeaw^9^, Alisa Tubsuwan^2^ and Kanit Bhukhai^1*^

^1^Department of Physiology, Faculty of Science, Mahidol University, Bangkok 10400, Thailand.

^2^Stem Cell Research Group, Institute of Molecular Biosciences, Mahidol University, Nakhon Pathom 73170, Thailand.

^3^Center of Multidisciplinary Technology for Advanced Medicine (CMUTEAM), Faculty of Medicine, Chiang Mai University, Chiang Mai 50200, Thailand.

^4^Chakri Naruebodindra Medical Institute, Faculty of Medicine Ramathibodi Hospital, Mahidol University, Samut Prakan, Thailand, 10540, Thailand.

^5^Prima Scientific, 147/170-171 Baromrajchonnee, Arunamarin, Bangkok 10700, Thailand.

^6^Department of Chemistry and Center of Excellence for Innovation in Chemistry, Faculty of Science, Ramkhamhaeng University, Bangkok 10240, Thailand.

^7^Center for Scientific Instrumentation and Platform Services Unit, Faculty of Science, Mahidol University, Bangkok 10400, Thailand.

^8^Department of Pediatrics, Faculty of Medicine, Ramathibodi Hospital, Mahidol University, Bangkok 10400, Thailand.

^9^Siriraj Integrative Center for Neglected Parasitic Diseases, Department of Parasitology, Faculty of Medicine Siriraj Hospital, Mahidol University, Bangkok 10700, Thailand.

*Corresponding author: Kanit Bhukhai

kanit.bhu@mahidol.ac.th; kanitscmu@gmail.com

Department of Physiology, Faculty of Science, Mahidol University, Bangkok 10400, Thailand.

**Supplemental experiment procedures**

**May Grunwald-Giemsa staining for morphological observation**

hiPSC-derived CD34^+^ cells, CFUs/BFUs of blood-differentiating cells, and cultured hiPSC-derived erythroid cells were collected and resuspended in 100 μL of DPBS. Following the manufacturer's procedure of cytospin, the cell suspension was loaded into a cytoblock cassette and spun at 600 rpm for two min. After centrifugation, the cells were attached to the glass slide and air-dried before being fixed with May-Grunwald dye for four minutes. Before staining, Giemsa dye was freshly diluted with tap water with a ratio of a droplet of dye per 1 mL of water. The slides were treated with the diluted Giemsa for 40-45 minutes. Stained slides were rinsed with tap water and air-dried. Then, the slides were cleaned with absolute methanol to observe under the bright-field microscope.

**Gene expression analysis using NanoString nCounter^®^ Stem Cell panel and assay**

The modulation of gene expression in distinct cell conditions was determined by NanoString nCounter Technology using the nCounter® Stem Cell Characterization Panel (XT Hs StemCell CSO-12, NanoString Technologies) with multiplex gene expression analysis of 770 genes. As a preparation step, the mRNA probe hybridization of 100 ng total RNA of four samples from each condition, including hiPSC-derived HSPCs (hiPS-HSPC), hiPS-HSPC + ASPP 049, and CD34^+^ umbilical cord blood (UCB) was performed at 65 °C for 18 hours. The samples were then loaded on the nCounter SPRINTTM Cartridge (NS-100078, NanoString Technologies) according to the NanoString sample preparation instructions (https://www.nanostring.com/ support/product-support/support-documentation). Processing on the nCounter Digital Analyzer, high sensitivity mode was selected on the nCounter Prep station. This station specifies the number of images (fields of view) for analysis per assay, corresponding to the number of collected data from the option of FOV 555 mode. Then, the machine scored the number of fluorescence barcodes of each gene. Data from the nCounter system exported as RCC files were evaluated by nSolver^TM^ software, v4.0 analysis. The transcript copies were normalized using the geometric means of 12 housekeeping genes for the individual conditions. Moreover, the geometric means of negative control threshold count values were used for background subtraction. The fold changes in gene expression profiling were indicated by comparing each condition. Raw p-values and false discovery rates were used to assess the significance of the data analysis.

**The** **erythroid induction from hiPS-HSPCs**

To further confirm the function, for instance, cells underwent erythroid differentiation. hiPSC-derived CD34^+^ nonadherent cells were harvested and magnetically isolated as described in the method. Subsequently, the cells were seeded at a density of 2 × 10^5 cells per mL in a three-stage erythroid culture, as previously outlined **[1, 2]**. As shown in Fig. S2A, the basal medium consists of Iscove’s Modified Dulbecco’s Medium (IMDM, Hyclone), supplemented with 3% human AB serum (Atlantis Biosciences), 2% Fetal Bovine Serum (Hyclone), 0.2 mg/mL holo-transferrin (holoTf, Sigma), 3 U/mL heparin (Sigma), 10 μg/mL human insulin (Sigma), and 3 U/mL Erythropoietin (EPO, HEMAX). In the first stage, on days 0E to 8E, the basal medium was supplemented with cytokines (all from Peprotech) at concentrations of 10 ng/mL SCF and 1 ng/mL IL-3. In the next stage, on days 8E to 11E, the medium contained 10 ng/ml SCF and an additional 0.5 mg/mL holoTf (up to 0.8 mg/mL). The final stage, on days 11E to 15E, involved an additional 0.5 mg/mL holoTf.

**Flow cytometry analysis for erythroid differentiation**

At evaluated time points, differentiating cells were harvested, pelleted, and stained with antibodies in PBE buffer (PBS + 0.1% BSA + 2 mM EDTA). The antibodies used to assess erythroid surface markers were PE-conjugated CD71 (Miltenyi) and PEcy7-conjugated CD235a (BioLegend). Detailed information on these antibodies is provided in Additional file: Table S1. The stained erythroid cells were detected using the BD FACSCelesta^TM^ Cell analyzer (BD Biosciences), and the data were analyzed using Flowjo software.

**RNA preparation and** **quantitative real-time PCR (qRT-PCR) analysis for the globin expression**

According to the manufacturer’s protocol, total RNA isolated from erythroid cells at evaluated days utilized TRIzol Reagent (Ambion). RNA was treated with DNase I (ThermoFisher Scientific) and then synthesized to cDNA using RevertAid First-Strand cDNA Synthesis Kit (ThermoFisher Scientific). The expression of mRNA was examined by 5-types of globin-specific oligonucleotides (Additional file 1: Table S2) using FastStart Essential DNA Green Master (Roche) on CFX96 Real-Time System (Bio-Rad) according to the manufacturer’s instructions. Containing the human ζ-, ⍺-, 𝛆-, 𝛾-, β- globin genes. A tandem subcloned DNA plasmid was used to generate standard curves for quantification of globin copy number as previously described [3]. The correlation coefficients, R-square, were not below 0.99 for standard curves and acceptable linear equations. The ratio of each globin was calculated using its copy number normalized by total ⍺- or β- like globin separately.

**Supplementary figures**

**
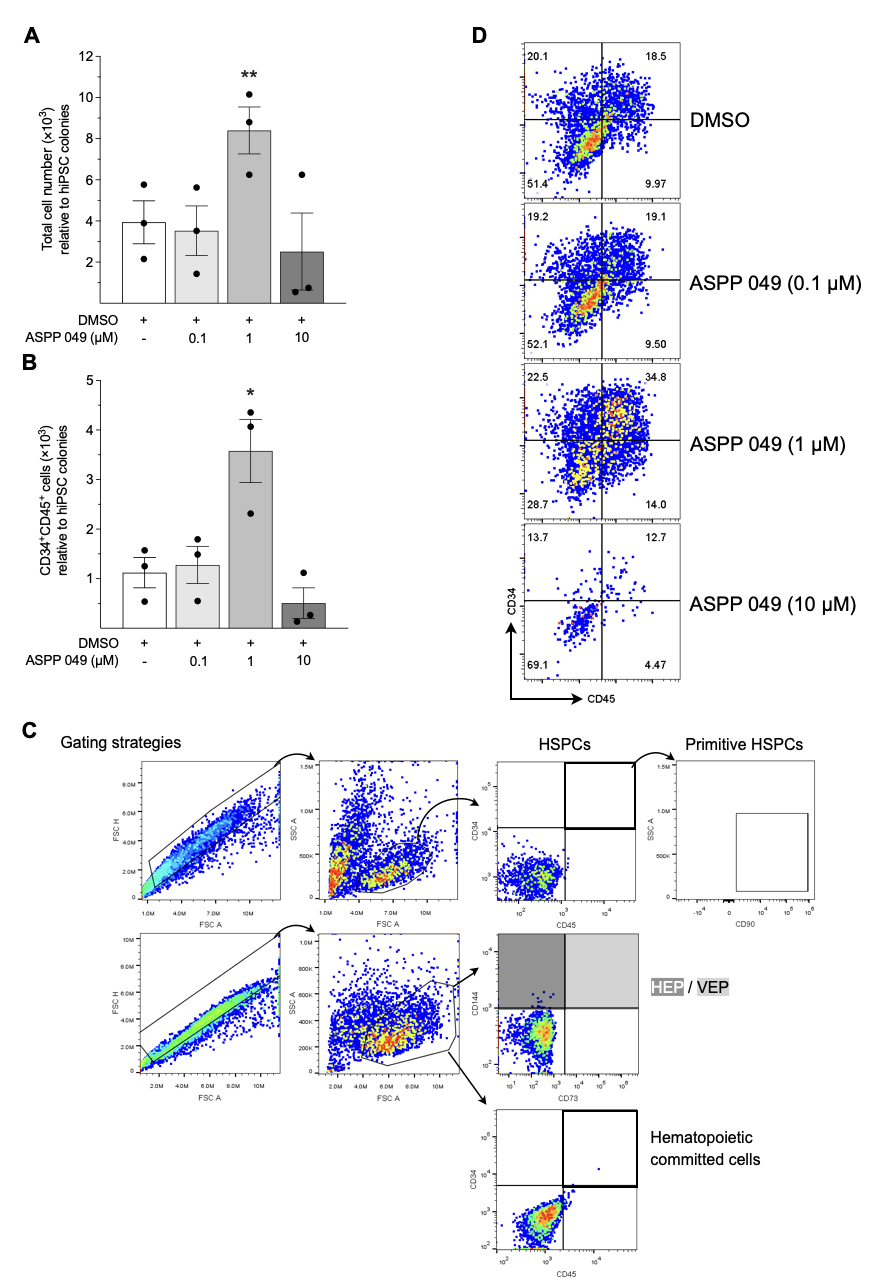
**

**Figure S1 Effects of ASPP 049 on nonadherent cells derived from hematopoietic differentiation.** After differentiation, day 12 nonadherent cells at different concentrations of ASPP 049 were tested by trypan blue exclusion assay and flow cytometry analysis, indicating **A** cell viability and **B** CD34^+^CD45^+^ HSPC number derived from hiPSCs. Individual data are represented as mean±SEM, with three biological replicates. The significance of *p-value < 0.05, **p-value < 0.01 was indicated by repeated-measures one-way ANOVA with Dunnett’s multiple comparisons. **C** Flow cytometric gating strategy for specifying HSPCs and primitive HSPCs. **D** Representative flow cytometric plots of HSPCs using surface markers CD34 and CD45.


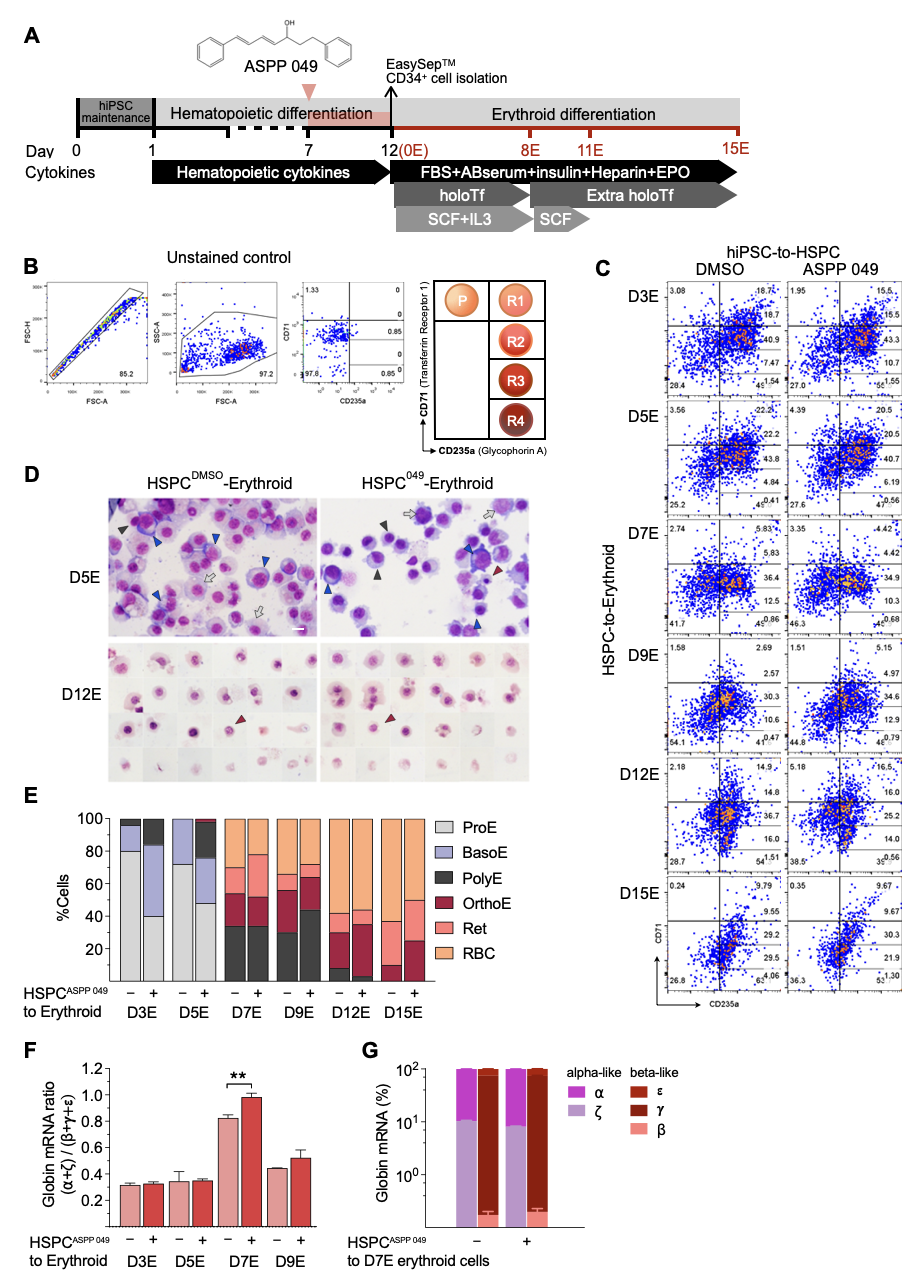


**Figure S2 The effects of ASPP 049 on hiPS-HSPCs toward erythroid differentiation.** Day 12 nonadherent cells from hematopoietic differentiation of both conditions were magnetically sorted for CD34^+^ seeded cells on day 0E. **A** Experimental schematics of three stages of erythroid differentiation. **B** Flow cytometric gating strategy for kinetic evaluation of erythroid surface markers (CD71, transferrin receptor 1 and CD235a, glycophorin A). **C** Representative flow cytometric plots of erythroid dynamic changes in every other differentiating day of HSPC without ASPP 049 (HSPC^DMSO^-to-erythroid) and HSPC with ASPP 049 (HSPC^049^-to-erythroid). **D** Representative morphologies: ProE (grey arrow), BasoE (blue arrowhead), PolyE (black arrowhead), OrthoE (red arrowhead) and **E** quantitative percentages of stained erythroid cells derived from hiPS-CD34^+^ HSPCs on determined days of differentiation culture. At least 100 cells were counted in each group. Abbreviations: ProE, proerythroblasts; BasoE, basophilic erythroblasts; PolyE, polychromatophilic erythroblasts; OrthoE, orthochromatic erythroblasts; Ret; reticulocytes, RBC; red blood cells, Scale bar = 50 μm. **F** Differential ratio of globin genes (ζ+⍺/𝛆+𝛾+β) on D7E erythroid cells derived from hiPS-HSPC^DMSO^ and HSPC^049^ measured by qRT-PCR. **G** Percentage of each globin mRNA expression: HBZ (ζ/ ζ+⍺), HBA (⍺/ ζ+⍺), HBE (𝛆/ 𝛆+𝛾+β), HBG (𝛾/ 𝛆+𝛾+β), HBB (β /𝛆+𝛾+β) from different erythroid conditions. Proportions of ⍺- and β-like globin mRNA can indicate globin-programmed switching. Data are represented as mean±SEM from three technical replicates, **p < 0.01, compared with HSPC-to-erythroid control (unpaired student's *t*-test).


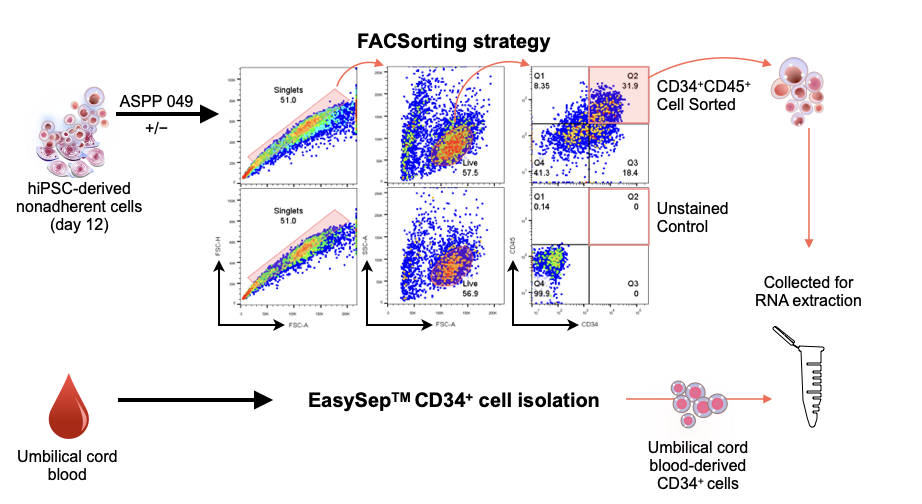
 **Figure S3 Illustrations of cell specification** using the gating strategy for fluorescence-activated cell sorting (FACS) of hiPSC-derived CD34^+^CD45^+^ HSPCs and magnetically sorting umbilical cord blood-derived CD34^+^ cells.


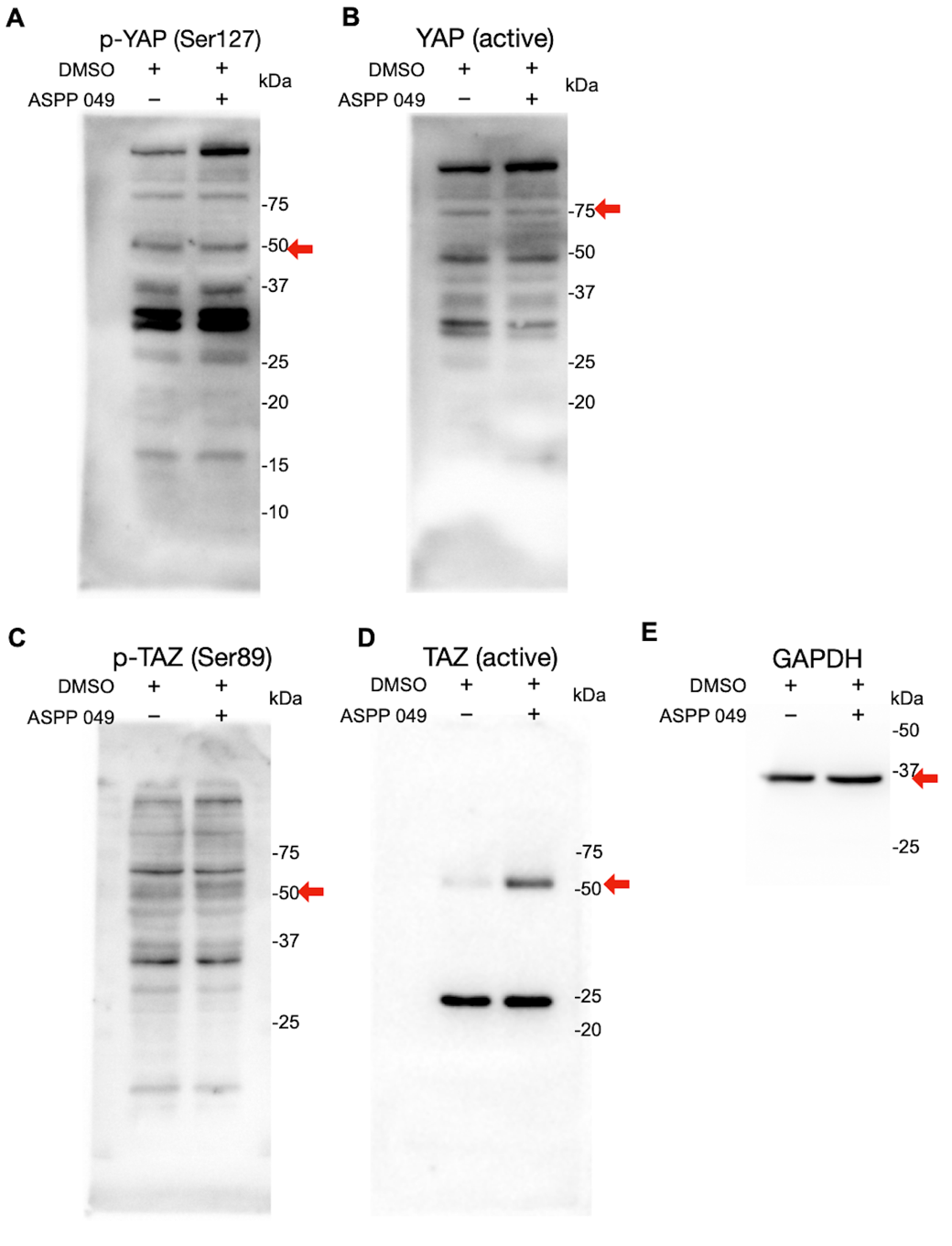


**Figure S4 Full-length Immunoblotting** of hiPSC-derived CD34^+^ cells on day 12 hematopoietic differentiation with and without ASPP 049 supplementation probed with **A** anti-p-YAP (predicted molecular weight 54 kDa), **B** anti-YAP (predicted molecular weight 75 kDa), **C** anti-p-TAZ (predicted molecular weight 35-50 kDa), **D** anti-TAZ (predicted molecular weight 55 kDa), and **Es** anti-GAPDH (predicted molecular weight 37 kDa).


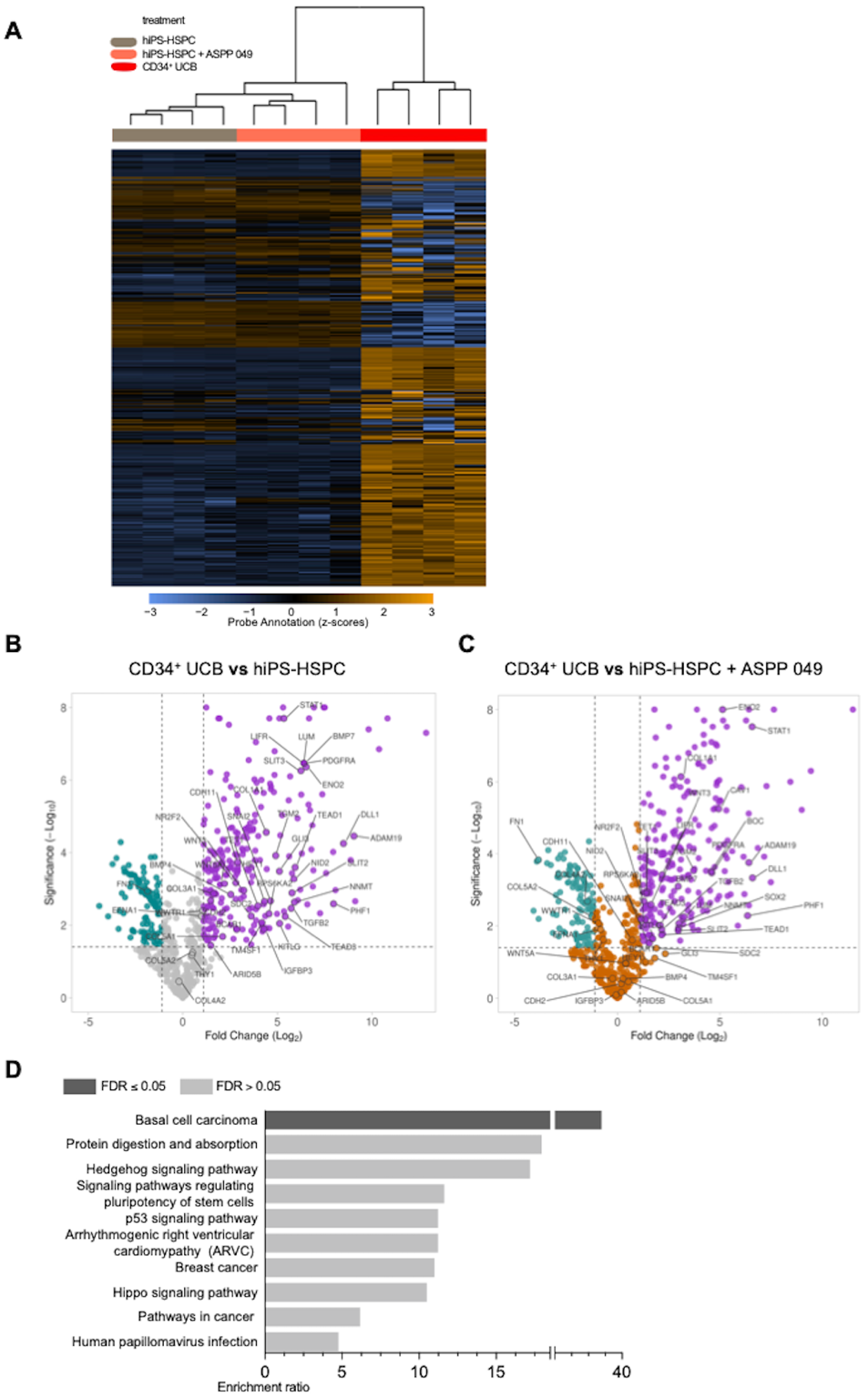


**Figure S5 ASPP 049 treatment induces the rescue pattern of gene expression within hiPS-HSPC that involves stemness similar to CD34^+^ UCB. A** Unsupervised heatmap obtained with sample cluster analysis of gene expression profiles between hiPS-HSPC, hiPS-HSPC + ASPP 049, and CD34**^+^** UCB. **B** Volcano plot represents the differentially expressed genes (DEGs) of CD34^+^ UCB compared to hiPS-HSPC and **C** CD34^+^ UCB compared to ASPP 049-treated group. **D** Pathways enrichment ratio determined by 11 genes of interest showed that the Hippo signaling pathway consistently correlates with 46 DEG. Individual data of expression levels is represented from four biological replicates as a color ranging of mRNA expression levels: blue, downregulated; black, no difference; yellow, upregulated.

**
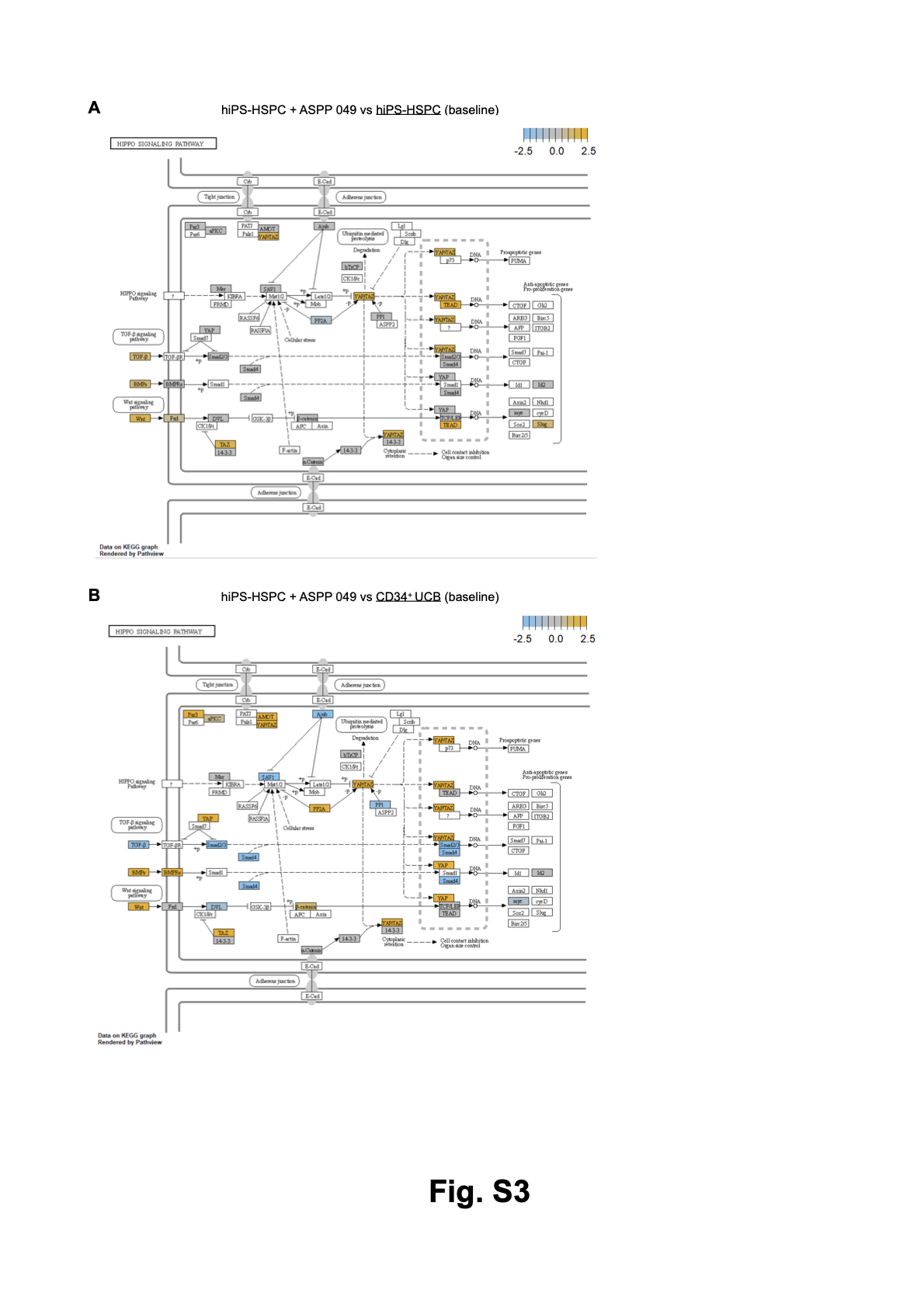
**

**Figure S6 Prediction of protein-based KEGG analysis overlay the pathway and differential expression information from ASPP049 effects** KEGG Pathview mapped pathway and DEGs significantly correlated to the protein-coding genes involved with Hippo signaling pathway between **A** hiPS-HSPC + ASPP 049 vs hiPS-HSPC (baseline) and **B** hiPS-HSPC + ASPP 049 vs CD34^+^ UCB (baseline), White nodes; no genes in Nanostring mRNA analysis, grey nodes; corresponding genes in the analysis with no significant differences, Blue nodes; downregulated genes relative to the baseline, Yellow nodes: upregulated genes relative to the selected baseline. The differences in signal levels were obtained from four biological replicates of NanoString^®^ mRNA analysis.

**Supplementary table**


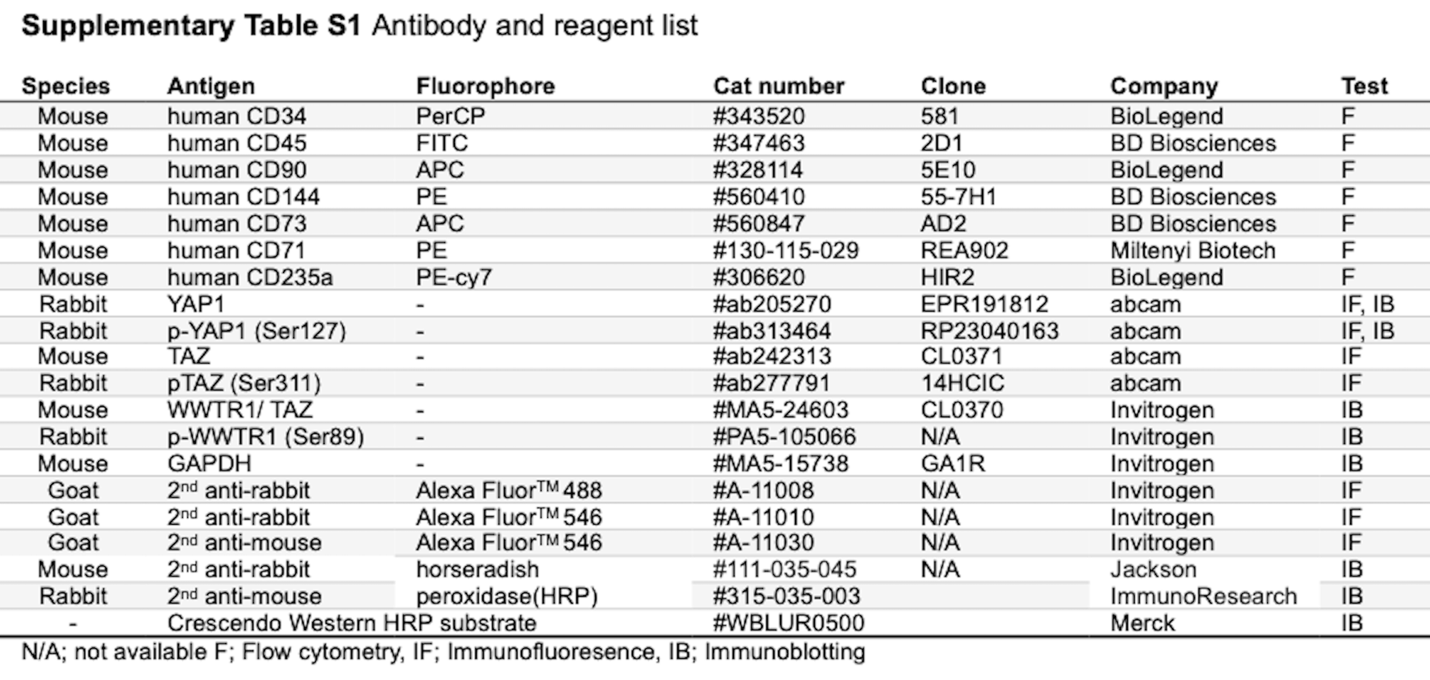


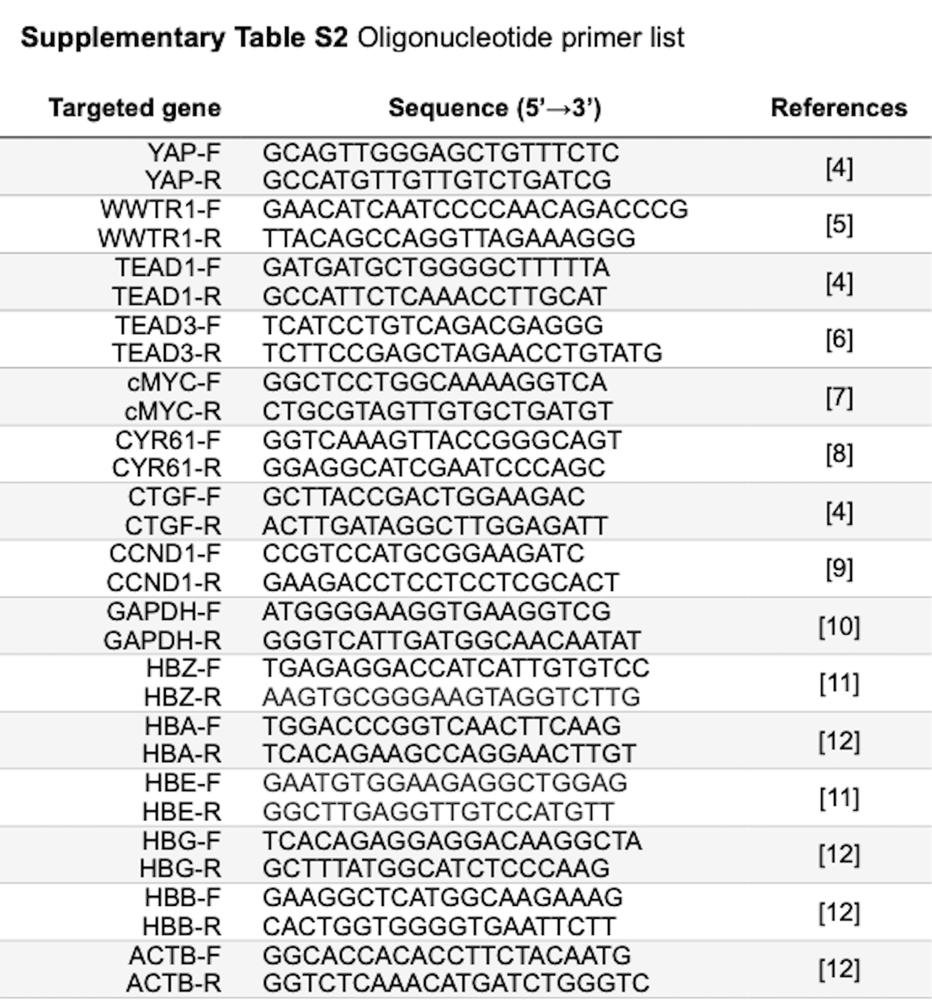


**
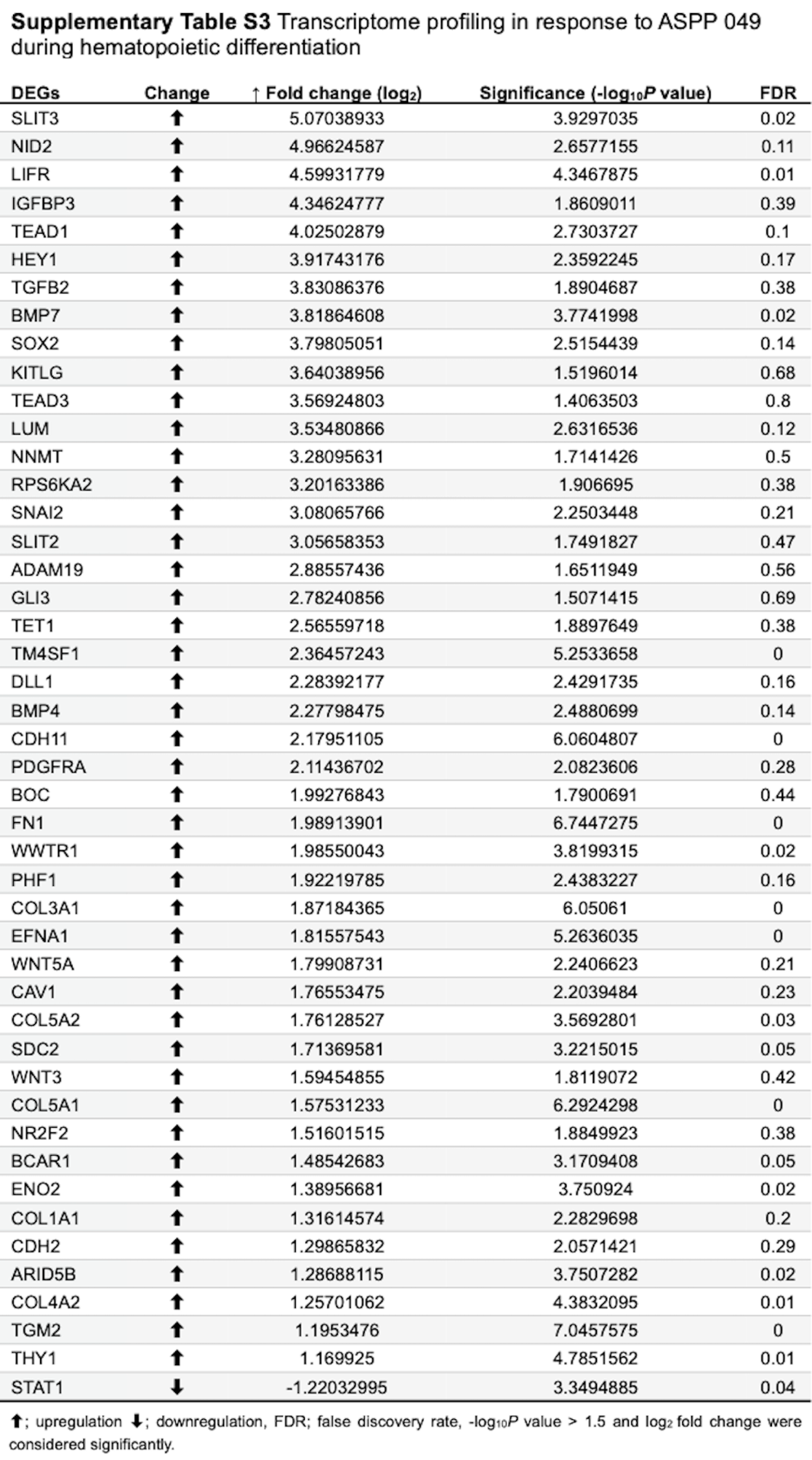
**

**T**he changes and significance of 46 differentially expressed genes determined by bulk Nanostring mRNA analysis of CD34^+^CD45^+^ sorted cells in the presence of ASPP 049 (1 µM) compared with the absence. Four replicates were performed for sampling from each condition.

**
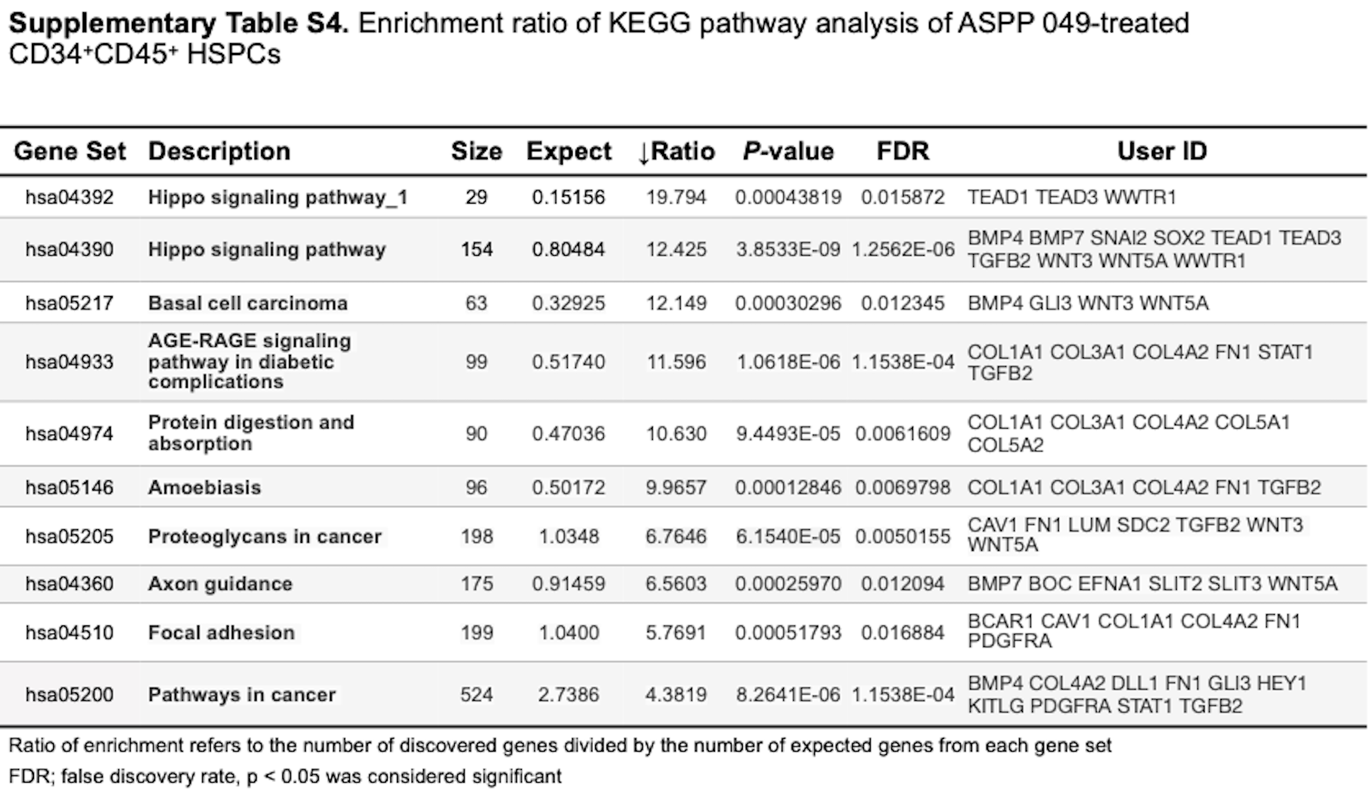
**

KEGG pathway analysis was performed using the WebGestalt online tool. 46 differentially expressed genes were classified into each gene set, highly related to the Hippo signaling pathway, FDR < 0.05, and enrichment ratios = 19.794 and 12.425.


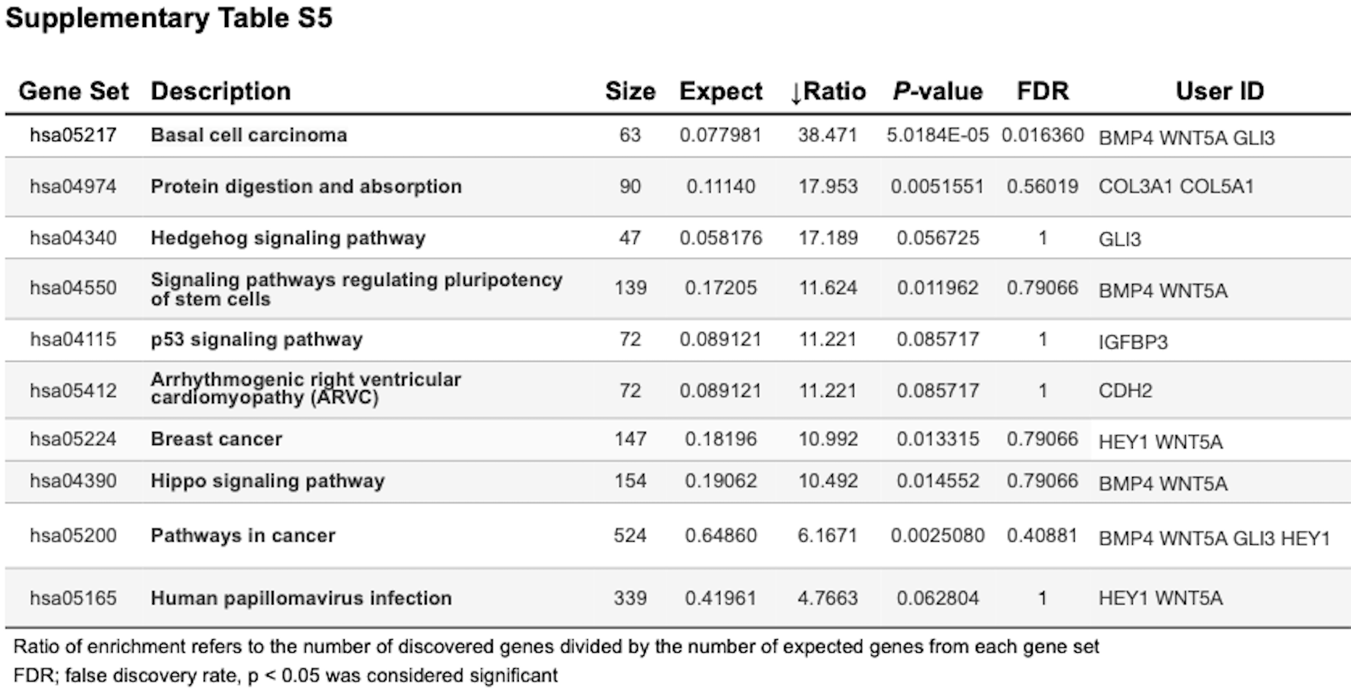


KEGG pathway analysis was performed using the WebGestalt online tool. 11 genes of interest were retrieved similar to CD34^+^ UCB and significantly classified into each gene set of related pathways. Although the most significant gene set was the basal cell carcinoma, the Hippo signaling pathway (FDR > 0.05) was still found to correlate between hiPS-HSPC + ASPP 049 and CD34^+^ UCB with enrichment ratios = 10.492.

**Supplementary references**

1. Griffiths RE, Kupzig S, Cogan N, Mankelow TJ, Betin VM, Trakarnsanga K, et al. Maturing reticulocytes internalize plasma membrane in glycophorin A-containing vesicles that fuse with autophagosomes before exocytosis. Blood. 2012;119(26):6296-306.

2. Trakarnsanga K, Ferguson D, Daniels DE, Griffiths RE, Wilson MC, Mordue KE, et al. Vimentin expression is retained in erythroid cells differentiated from human iPSC and ESC and indicates dysregulation in these cells early in differentiation. Stem Cell Res Ther. 2019;10(1):130.

3. Tubsuwan A, Abed S, Deichmann A, Kardel MD, Bartholoma C, Cheung A, et al. Parallel assessment of globin lentiviral transfer in induced pluripotent stem cells and adult hematopoietic stem cells derived from the same transplanted beta-thalassemia patient. Stem Cells. 2013;31(9):1785-94.

4. Luo H, Yu Q, Liu Y, Tang M, Liang M, Zhang D, et al. LATS kinase-mediated CTCF phosphorylation and selective loss of genomic binding. Sci Adv. 2020;6(8):eaaw4651.

5. Yu J, Alharbi A, Shan H, Hao Y, Snetsinger B, Rauh MJ, et al. TAZ induces lung cancer stem cell properties and tumorigenesis by up-regulating ALDH1A1. Oncotarget. 2017;8(24):38426-43.

6. Park S, Mossmann D, Chen Q, Wang X, Dazert E, Colombi M, et al. Transcription factors TEAD2 and E2A globally repress acetyl-CoA synthesis to promote tumorigenesis. Mol Cell. 2022;82(22):4246-61 e11.

7. Choi W, Kim J, Park J, Lee DH, Hwang D, Kim JH, et al. YAP/TAZ Initiates Gastric Tumorigenesis via Upregulation of MYC. Cancer Res. 2018;78(12):3306-20.

8. Wang H, Zhang S, Zhang Y, Jia J, Wang J, Liu X, et al. TAZ is indispensable for c-MYC-induced hepatocarcinogenesis. J Hepatol. 2022;76(1):123-34.

9. Ou WB, Ni N, Zuo R, Zhuang W, Zhu M, Kyriazoglou A, et al. Cyclin D1 is a mediator of gastrointestinal stromal tumor KIT-independence. Oncogene. 2019;38(39):6615-29.

10. Raimondo S, Naselli F, Fontana S, Monteleone F, Lo Dico A, Saieva L, et al. Citrus limon-derived nanovesicles inhibit cancer cell proliferation and suppress CML xenograft growth by inducing TRAIL-mediated cell death. Oncotarget. 2015;6(23):19514-27.

11. Tangprasittipap A, Kaewprommal P, Sripichai O, Sathirapongsasuti N, Satirapod C, Shaw PJ, et al. Comparison of gene expression profiles between human erythroid cells derived from fetal liver and adult peripheral blood. PeerJ. 2018;6:e5527.

12. Nualkaew T, Khamphikham P, Pongpaksupasin P, Kaewsakulthong W, Songdej D, Paiboonsukwong K, et al. UNC0638 induces high levels of fetal hemoglobin expression in beta-thalassemia/HbE erythroid progenitor cells. Ann Hematol. 2020;99(9):2027-36.
